# Estimating Environmental RF‐EMF Exposure From Mobile Networks Using Signal Indicators Collected With the ETAIN 5G Scientist App

**DOI:** 10.1002/bem.70071

**Published:** 2026-10-13

**Authors:** Nekane Sandoval‐Diez, Lea Belácková, Hamed Jalilian, Adriana Fernandes Veludo, Lloyd Roga, Isabelle Deltour, Stuart Henderson, Sarah Loughran, Arno Thielens, Marco Zahner, Jürg Fröhlich, Anke Huss, Martin Röösli

**Affiliations:** ^1^ Swiss Tropical and Public Health Institute Allschwil Basel‐Land Switzerland; ^2^ University of Basel Basel Basel City Switzerland; ^3^ Institute for Risk Assessment Sciences Utrecht University Utrecht Utrecht the Netherlands; ^4^ International Agency for Research on Cancer Lyon Auvergne‐Rhône‐Alpes France; ^5^ Australian Radiation Protection and Nuclear Safety Agency Yallambie Victoria Australia; ^6^ ASRC, The Graduate Centre of the City University of New York New York City New York USA; ^7^ Fields at Work LLC Zurich Zurich Switzerland

**Keywords:** measurement error, population‐level exposure assessment, smartphone‐based measurements

## Abstract

Smartphones track indicators of a reference signal to maintain network connectivity. These can serve as proxies for a subset of environmental radiofrequency electromagnetic field (RF‐EMF) exposure from mobile networks. In this multi‐country study, we developed conversion functions linking signal indicators collected with the ETAIN 5G‐Scientist app to measured RF‐EMF exposure. Parallel measurements were conducted across six European countries and Australia using frequency‐selective exposimeters and the app. Associations of signal strength and quality indicators with exposure were analysed using log‐linear regression models: (1) serving‐cell level, relating exposure in the serving frequency band to indicators of the connected cell (Europe *n* = 2960; Australia *n* = 747); and (2) location level, relating indicators aggregated across network providers to environmental exposure from 4G‐LTE and 5G‐NR networks (Europe *n* = 61; Australia *n* = 48). At the serving‐cell level, 4G‐LTE signal strength was positively associated with exposure, while signal quality was inversely associated. For 5G‐NR, only signal strength was associated with exposure, with weaker and less consistent relationships than for 4G‐LTE. At location level, aggregation across providers reduced model error, suggesting residual variability operates at short temporal and spatial scales. These findings support using smartphone signal indicators to estimate components of environmental RF‐EMF exposure, although further validation is needed, particularly for 5G‐NR. Bioelectromagnetics. 00:00–00, 2026. © 2026 Bioelectromagnetics Society.

## Highlights

- Serving‐cell 4G‐LTE signal strength showed strong positive associations with serving‐band environmental RF‐EMF exposure (*β* = 0.74 in Europe; *β* = 0.62 in Australia).
- Serving‐cell 5G‐NR signal strength showed moderate associations with serving‐band environmental RF‐EMF exposure (*β* = 0.56 in Europe; *β* = 0.41 in Australia).
- Aggregation of signal indicators across network providers improved model performance and strengthened the association between 4G‐LTE signal indicators and environmental exposure (*β* = 0.86 in Europe and *β* = 0.82 in Australia; adjusted *R* ^2^ up to 0.91 vs ≤ 0.63 for serving‐cell models).

## 1 Introduction

Given the widespread deployment and ongoing evolution of mobile telecommunication networks (David and Berndt 2018), there is a need for transparent information on population exposure to radiofrequency electromagnetic fields (RF‐EMF). Characterisation of typical exposure levels is essential to inform the public and support epidemiological research assessing potential health effects. Meeting this need requires exposure assessment approaches that can capture real‐world variability across space, time, and network conditions.

Characterising RF‐EMF exposure is challenging because field strength varies strongly in space and time (Röösli et al. 2010). Exposure can change by orders of magnitude over short distances due to spatial variability in propagation conditions (Aerts et al. 2023; Bornkessel et al. 2007; Heliot et al. 2022). In addition, network traffic and utilisation, and human behaviour contribute to substantial variability in exposure levels (Frei et al. 2009; Joseph and Verloock 2010; Velghe et al. 2019). Although dedicated measurement instruments, including personal exposimeters, provide accurate and validated measurements, their cost, logistical complexity, and participant burden limit their applicability for continuous exposure assessment over long time periods and across large geographic areas and population groups (Bhatt et al. 2016).

Smartphones may offer a complementary approach to exposure assessment as devices that continuously monitor signal strength and quality indicators to maintain network connectivity (Schießl 2023a). These signal strength and quality indicators, which are derived from downlink transmissions from serving base stations, reflect a component of the underlying environmental RF‐EMF exposure from mobile networks and may therefore serve as proxies for it, as they originate from the same source. With about 4.4 billion people worldwide owning a smartphone (GSMA 2025), such data can be collected via downloadable applications with minimal user burden, allowing scalable data collection. Although smartphones are not designed as dedicated measurement instruments and are not suitable for regulatory compliance monitoring (Fröhlich et al. 2016; Schießl 2023b), previous work has shown that they can provide reasonably accurate estimates of the LTE (Long Term Evolution) contribution to RF‐EMF exposure (Schießl 2023a). These estimates can support the development of exposure maps for risk communication and research (Schießl et al. 2021; López‐Espí et al. 2024).

Within the framework of the European Union Horizon‐funded ETAIN (Exposure To electromAgnetic fIelds and plaNetary health) project, we developed an open‐access Android application, the ETAIN 5G Scientist app (2025; Fields at Work 2025). The app collects mobile network signal indicators and provides users with information on their RF‐EMF exposure from personal mobile phone use and from mobile network base stations of both the user's own network provider and other providers. The aim of this paper is to derive and evaluate empirical conversion functions describing the association between smartphone‐recorded signal indicators and measured environmental RF‐EMF exposure from mobile networks, as a basis for future estimation of real‐world exposure.

## 2 Materials and Methods

To relate smartphone‐recorded signal indicators to measured environmental RF‐EMF exposure, we developed two complementary types of conversion functions reflecting different analytical perspectives and intended applications. First, we derived technical conversion functions (see Section 2.5.1), which model the association between signal indicators from the serving cell and measured environmental RF‐EMF exposure in the frequency band of the serving cell. The technical functions are implemented in the ETAIN 5G Scientist app to provide users with information on environmental exposure from their own network provider. Second, we derived map conversion functions (see Section 2.5.2), which model aggregated signal indicators across all detected network providers and the average environmental RF‐EMF exposure from LTE (Long Term Evolution) or NR (New Radio) networks at each location. The map functions are implemented to develop exposure maps (ETAIN exposure maps 2026).

The detailed measurement protocol has been described previously in Sandoval‐Diez et al. (2025). Here, we summarise the components relevant to this paper. The protocol was tested in one French city (Lyon) in July 2023 and subsequently implemented in four additional European countries (the Netherlands, Switzerland, Spain, and Greece) between October 2023 and November 2024. After standalone 5G (5G‐SA) services became available in Germany in early 2025 (European Commission 2025), we conducted an additional measurement campaign in one German city in April 2025. Because 5G‐SA has been widely deployed in Australia (Australian Competition and Consumer Commission 2025), a final campaign was conducted along the East Coast of Australia between July and August 2025.

### 2.1 Measurement Locations

In each country, we selected measurement locations to represent different levels of urbanicity and population density, which are associated with RF‐EMF exposure (Veludo et al. 2025). We included up to two big cities (> 500,000 inhabitants), up to two medium‐sized cities (100,000– 500,000 inhabitants), and two to four rural towns (< 100,000 inhabitants) in each country. In France and Germany, only one medium‐sized city was included. To capture different network loads and propagation conditions, measurement locations were distributed across different microenvironments (Tables S1–S7). These included city or town centres and residential areas, commercial or industrial areas, train stations, and green areas. For Australian locations, line‐of‐sight (LoS) versus non‐line‐of‐sight (NLoS) antenna situations were also documented (Table S8).

### 2.2 Exposure Assessment Tools

We measured RF‐EMF exposure using personal frequency‐selective exposimeters ExpoM‐RF4 (Fields at Work 2020), which record root mean square (RMS) values of electric field strength (in Vm^−1^) across predefined frequency bands from 50 MHz to 6 GHz. Devices were configured with a detection range up to 6 Vm^−1^, and customised frequency bands were programmed to reflect the mobile telecommunication frequency ranges currently used in Europe (Table S9) and Australia (Table S10). As this study focused on environmental RF‐EMF exposure originating from telecommunication networks, only frequency division duplexing (FDD) downlink (DL) and time division duplexing (TDD) bands were analysed. ExpoM‐RF4 devices perform 50‐ms sequential sweeps across all bands, which correspond to sampling intervals of 7‐s in Europe and 6‐s in Australia. In Germany and Australia (where 5G‐SA was available), a second ExpoM‐RF4 programmed only with 3.5GHz TDD bands (sampling interval of 2‐s) was used to approximate the environmental component of exposure in the 3.5GHz TDD bands (see Section 2.3).

The ETAIN 5G Scientist app (Fields at Work 2025) was used to collect mobile network parameters from the serving LTE or NR cell (i.e., the cell currently providing the connection) and up to nine neighbouring cells (nearby cells detected by the device but not actively serving the connection) from the same provider. The app logs data every second, together with a timestamp. Recorded variables included the carrier frequency, frequency band, signal strength and signal quality indicators. Frequency bands are reported using the standardised 3GPP band numbering system, where each band number corresponds to a defined operating frequency range for LTE or NR (3GPP (2025c; 2025d). Signal strength indicators (in dBm) included the Received Signal Strength Indicator (RSSI, for LTE only) and Reference Signal Received Power (RSRP). LTE‐RSSI represents the linear average of the total received power within the measurement bandwidth, including interference and thermal noise (3GPP 2025a). RSRP refers to the power of the cell‐specific reference signals for LTE (LTE‐RSRP) or the secondary synchronisation signals for NR (NR‐ssRSRP) (3GPP 2025b). Signal quality (in dB) was quantified using the LTE Reference Signal Received Quality (LTE‐RSRQ) and its NR counterpart (NR‐ssRSRQ). RSRQ is defined as the ratio between the reference signal (RSRP or ssRSRP) and the carrier total received power (RSSI), multiplied by the number of resource blocks (3GPP 2025a).

Recordings from the ETAIN 5G Scientist app were collected using Android (version 13) smartphones. Models used included Samsung S22 + , Samsung A22, Redmi Note 11 s, and Oppo A77.

### 2.3 Measurement Procedure and Setup

Spot measurements consisted of parallel recordings with the ExpoM‐RF4 and the ETAIN 5G Scientist app during predefined phone activities. To obtain data from all network providers, each smartphone was sequentially equipped with SIM cards from the major national providers at each measurement location, except in Germany, where measurements were conducted with a SIM card from one national provider only (Table S11). The main ExpoM‐RF4 device was mounted on a tripod at ~1.5 m height and ~30 cm from the phone. In Germany and Australia, a second ExpoM‐RF4 was placed ~1.5 m from the phone in the assumed direction of the serving beam (i.e., along the line between the phone and the serving base‐station antenna) to approximate environmental exposure in the 3.5GHz bands by reducing the contribution from the phone's uplink transmission. Antenna position was determined using publicly available antenna databases for Germany (Bundesnetzagentur 2026) and Australia (Australian Mobile Telecommunications Association 2026); because listed antenna coordinates may be subject to positional uncertainty, the serving‐beam direction was considered approximate. Each activity was carried out for 2 min and included: non‐use (idle mode), native voice calls, WhatsApp voice calls, WhatsApp video calls, and continuous file upload to an FTP server. During voice call activities, the phone was pressed against the ear, whereas for all other activities it was held in the hand in front of the body (Sandoval‐Diez et al. 2025). Not all activities were performed in all countries due to logistical and protocol refinements (Table S12) (Sandoval‐Diez et al. 2025). A custom‐developed, dedicated smartphone application was used to control the activities execution and register timestamps for synchronisation with the ETAIN 5G Scientist app and ExpoM‐RF4 logs.

### 2.4 Data Processing

#### 2.4.1 Crosstalk Correction

To reduce crosstalk‐related overestimation of measured exposure in closely spaced frequency bands, ExpoM‐RF4 measurements were corrected following the methodology described by Eeftens et al. (2018). This approach assumes that power contributions in adjacent frequency bands are uncorrelated and was applied to the band pairs below 3 GHz listed in Table S13.

One reason for the presence of crosstalk is that the effective bandwidth of the ExpoM‐RF4 filters is wider than the programmed nominal bandwidth, meaning adjacent bands can overlap even if nominally separated. Thus, the effective 3 dB bandwidths of 3.5GHz bands that were nominally defined as having 75 and 100 MHz bandwidth, had 79 and 124 MHz effective bandwidth, respectively. In Europe, the 3.5GHz bands programmed in the ExpoM‐RF4 were spaced 30 MHz apart to reduce this crosstalk (Table S9). In contrast, the corresponding Australian 3.5GHz bands were adjacent, leading to effective overlap (Table S10). This overlap implies correlated power across adjacent bands, for which the correction methodology by Eeftens et al. (2018) is not appropriate. Overlapping edges resulted in an estimated 62 MHz of duplicated measurement. Assuming an equal power distribution across the bands, this represents an overestimation of about 16.5%, which was applied as a correction factor to the aggregated 3.5GHz Australian measurements.

#### 2.4.2 Data Cleaning

Data from the ExpoM‐RF4 and the ETAIN 5G Scientist app were synchronised using timestamps, and analyses were restricted to paired observations. Only recordings where the ETAIN 5G Scientist app reported a serving LTE or NR cell were considered for analyses; observations from older technologies (WCDMA or GSM) were excluded (16,046 [5.3%] in Europe; 50 [0.1%] in Australia).

NR recordings in the 3.5GHz TDD bands during active use (i.e., activities other than non‐use) were excluded unless parallel measurements from the second ExpoM‐RF4 device were available. During non‐use, uplink transmission from the phone is likely minimal and measurements in the TDD bands from the main exposimeter approximate environmental exposure. In contrast, during active use, measurements in the TDD bands from the main exposimeter may include contributions from the phone's uplink transmission; therefore, only recordings with the second exposimeter, positioned further from the phone to reduce uplink contributions, were retained to approximate the environmental component of exposure in the 3.5GHz TDD bands (23,714 [83.7%] in Europe; 0 [0.0%] in Australia; Table S14).

LTE‐RSSI recordings with the Samsung S22+ were excluded due to a device‐specific artefact, apparent from a difference in cumulative distribution function compared to other phones (Figure S1).

#### 2.4.3 Normalisation of Signal Strength Indicators

For a given incident electric field strength, signal strength indicators vary with operating frequency due to the frequency dependence of the effective aperture of the receiving antenna, which scales inversely with wavelength (Balanis 2016; Korkmaz et al. 2024). To make signal strength indicator values comparable across frequency bands, we normalised them to a reference frequency of 1800 MHz, selected as a midpoint of the range used for LTE and NR networks. We adjusted the recorded LTE‐RSRP, LTE‐RSSI, and NR‐ssRSRP values (in dBm) by adding 20 log_10_ of the ratio between the operating frequency and the reference frequency (Sandoval‐Diez et al. 2025). This normalisation was applied prior to all subsequent computations.

#### 2.4.4 Data Aggregation

Before aggregation, all observations were transformed to linear units. Signal strength and quality indicators were transformed from dBm or dB to a linear scale (Box S1), and electric field strength values were converted to power flux density (Box S2). After aggregation, values were back‐transformed to their original units.

We performed data aggregation separately for the technical and map conversion functions. For the technical conversion functions, measurements were temporally aggregated over 2‐min windows, each corresponding to a specific activity (Section 2.3). Within these windows, observations were subdivided into serving‐cell intervals, defined as contiguous periods during which the same serving LTE or NR cell was reported by the ETAIN 5G Scientist app. Intervals shorter than 7‐s were excluded, as no unique corresponding ExpoM‐RF4 measurement could be assigned (Europe *n* = 309 [9.5%], Australia *n* = 48 [6.0%]). For each serving‐cell interval, mean signal indicators from the serving cell were calculated (Box S3). Environmental exposure in the serving frequency band was defined as the mean measured exposure in the single DL or TDD frequency band whose predefined frequency range corresponded to that of the serving LTE or NR cell reported by the ETAIN 5G Scientist app (Box S4).

For the map conversion functions, aggregation was performed in three steps at each spot measurement location (Box S5). First, for LTE, signal strength indicators (RSRP or RSSI) were each summed across the serving and detected neighbouring cells at each recorded time point of the app, whereas signal quality (RSRQ) was averaged across these cells. For NR, only serving‐cell ssRSRP recordings were considered, as including ssRSRP from detected neighbouring NR cells did not result in a clearer positive correlation with measured environmental RF‐EMF exposure (Figure S2). Second, the resulting values were averaged across all recorded time points for each network provider at each location (i.e., across all activities) to obtain provider‐specific signal indicators. Third, these provider‐specific values were combined to obtain location‐level aggregates. Signal strength indicators (LTE‐RSRP, LTE‐RSSI, and NR‐ssRSRP) were each combined by summation across providers in each location, whereas signal quality indicators (LTE‐RSRQ and NR‐ssRSRQ) were each combined by averaging across providers in each location. Additionally, we derived composite location‐level signal metrics. Specifically, location‐level LTE‐RSRP and NR‐ssRSRP were summed to obtain a combined received power indicator across LTE and NR technologies, and location‐level LTE‐RSRQ and NR‐ssRSRQ were averaged to obtain a combined signal quality indicator across technologies.

Environmental RF‐EMF exposure from LTE network at each location was defined as the sum of DL and TDD bands below 2.6 GHz, measured with the ExpoM‐RF4 (Tables S9–S10, Box S6). Environmental RF‐EMF exposure from LTE and NR networks was defined as the sum of all measured mobile DL and TDD bands (Tables S9–S10, Box S6). Because these exposure metrics correspond to frequency‐band aggregates and could not be attributed to individual network providers, map conversion analyses were limited to locations where the measurement protocol covered all national providers. Therefore, German locations were excluded from these analyses because measurements there were conducted with only one national provider (Table S15). In Australia, measurements were planned for all three national providers at all locations; however, a small number of recordings failed for some providers at individual locations (Table S15). These failures were due to technical reasons and were not interpreted as an absence of network coverage. In addition, Australian locations were not included for NR map conversion functions because NR connectivity could only be established with one of the three national providers, although NR connectivity was confirmed on site and NR antenna availability was indicated by the antenna database (Australian Mobile Telecommunications Association 2026).

### 2.5 Data Analysis

To establish conversion functions, we fitted multivariable log‐linear regression models to estimate the associations of signal strength indicators (in dBm, proxies of propagation conditions) and signal quality indicators (in dB, proxies of network variability) with measured environmental RF‐EMF exposure. Measured exposure values (in Vm^−1^) were transformed to dBµVm^−1^ (Box S7) for analyses. The models were specified as:

(1) $$\mathrm{Exposure}=\beta_{0}+\beta_{1}S\mathrm{ignal}\;\mathrm{Strength}+\beta_{2}S\mathrm{ignal}\;\mathrm{Quality}+\epsilon$$

Where exposure denotes electric field strength (in dBµVm^−1^), $\beta_{0}$ is the intercept, $\beta_{1}$ and $\beta_{2}$ are regression coefficients representing the change in exposure per unit increase in signal strength (dBm) and signal quality (dB), respectively, and ε represents the random error term of each observation. The contribution of signal strength and signal quality indicators to the model fit was evaluated using likelihood ratio tests (LRT). Model performance and fit were assessed using the adjusted *R* ^2^ and the root mean square error.

Based on the data aggregation level, we developed a technical function (Section 2.5.1) and a map function (Section 2.5.2). All analyses were performed separately for Europe and Australia and stratified by mobile technology. LTE analyses included intervals where only LTE cells were reported and those where both LTE and NR cells were reported (5G non‐standalone, 5G‐NSA). NR analyses included intervals with NR‐only cells (5G standalone, 5G‐SA) as well as those with 5G‐NSA. All analyses were conducted in R (version 4.5.1) within RStudio (2025.5.1.513).

#### 2.5.1 Technical Conversion Functions

Technical conversion functions aimed to model the relationship between signal indicators from the serving cell and environmental RF‐EMF exposure in the serving frequency band. The unit of analysis was the aggregated signal indicators and exposure in serving‐cell intervals (see Section 2.4.4; Europe *n* = 2960, Australia *n* = 747). For LTE, both RSSI and RSRP were evaluated to compare their model performance.

To evaluate potential sources of uncertainty and systematic variation in exposure beyond that captured by the signal indicators, the base model (model 0, Equation 1) was extended by adding a set of additional network and contextual variables (model 1). The set of additional variables comprised network provider, serving frequency band, activity, smartphone model (Europe only, included to capture potential device‐specific differences in antenna characteristics, such as gain, polarisation, and radiation pattern), antenna situation (Australia only, NLOS vs LOS), indoor/outdoor setting (Europe only), microenvironment, urbanicity, and country (Europe only). Network provider was included as a random intercept to account for the multilevel data structure.

For mixed models, goodness of fit was assessed using marginal *R* ^2^ (i.e., variance explained by fixed effects) (Nakagawa et al. 2017). We quantified the relative contribution of each predictor using semi‐partial *R* ^2^ for fixed effects and the intraclass correlation coefficient (ICC) for random effects. Semi‐partial *R* ^2^ measures the unique variance explained by a predictor after accounting for its covariance with other variables, relative to the total variance in the outcome (Stoffel et al. 2021). Larger effect sizes (semi‐partial *R* ^2^ and ICC) indicated a higher potential for systematic prediction bias across levels of network and contextual variables. Multicollinearity was assessed using variance inflation factors, which showed low collinearity across predictors (range: 1.01‐2.33).

#### 2.5.2 Map Conversion Functions

Map conversion functions aimed to model the relationship between aggregated signal indicators across all detected network providers and the average environmental RF‐EMF exposure from LTE and NR networks at each location. Separate analyses were conducted for LTE and NR signal indicators. LTE signal indicators were related to average environmental RF‐EMF exposure from LTE networks, whereas NR signal indicators were related to average environmental RF‐EMF exposure from both LTE and NR networks, reflecting the shared use of frequency bands between these technologies. Given the inability to attribute measured exposure within shared bands to a specific technology, we additionally evaluated composite signal metrics combining received power and signal quality indicators across LTE and NR technologies (defined in Section 2.4.4). The unit of analysis was the location‐level aggregation of signal indicators and exposure (see Section 2.4.4; LTE: Europe *n* = 61, Australia *n* = 48; NR: Europe *n* = 48).

## 3 Results

A total of 2960 serving‐cell intervals were included in Europe and 747 in Australia, with mean durations of 94.1 ± 36.4 and 101.5 ± 30.3 s, respectively. Table 1 provides an overview of all serving‐cell intervals included in the technical function analyses. In Australia, 680 intervals corresponded to periods during which an LTE serving cell was reported, and 67 intervals (from a single network provider) corresponded to periods with a 5G‐SA NR serving cell. Only 12 of the NR serving‐cell intervals were associated with a 3.5GHz band, whereas the remainder corresponded to FDD bands. In Europe, most intervals corresponded to LTE serving cells (LTE‐only: 2710; 5G‐NSA: 187), whereas 250 intervals corresponded to NR serving cells (5G‐SA: 63; 5G‐NSA: 187).

**Table 1:** Characteristics of serving‐cell intervals included in the technical function analyses for Europe and Australia.

		Europe		Australia	
		LTE serving‐cell intervals *N* = 2897^a^	NR serving‐cell intervals *N* = 250^b^	LTE serving‐cell intervals *N* = 680	NR serving‐cell intervals *N* = 67
Urbanicity, *n* (%)					
Big city		1065 (36.8)	101 (40.4)	305 (44.9)	33 (49.3)
Medium‐sized city		1368 (47.2)	94 (37.6)	248 (36.5)	16 (23.9)
Town		464 (16.0)	55 (22.0)	127 (18.7)	18 (26.9)
Microenvironment, *n* (%)					
Centre or residential		1433 (49.5)	134 (53.6)	348 (51.2)	44 (65.7)
Industrial or commercial		521 (18.0)	37 (14.8)	152 (22.4)	10 (14.9)
Train station		494 (17.1)	47 (18.8)	42 (6.2)	3 (4.5)
Green area		449 (15.5)	32 (12.8)	138 (20.3)	10 (14.9)
Setting, *n* (%)					
Outdoor		1842 (63.6)	170 (68.0)	667 (98.1)	67 (100.0)
Indoor		1055 (36.4)	80 (32.0)	13 (1.9)	0 (0.0)
Phone, *n* (%)					
Samsung S22+		416 (14.4)	0 (0.0)	680 (100.0)	67 (100.0)
Samsung A22		1248 (43.1)	187 (74.8)	—	—
Redmi Note 11 s		625 (21.6)	30 (12.0)	—	—
Oppo A77		608 (21.0)	33 (13.2)	—	—
Activity, *n* (%)					
File upload		942 (32.5)	67 (26.8)	296 (43.5)	25 (37.3)
WhatsApp video call		649 (22.4)	48 (19.2)	—	—
WhatsApp voice call		622 (21.5)	16 (6.4)	—	—
Native voice call		408 (14.1)	0 (0.0)	194 (28.5)	0 (0.0)
Non‐use		276 (9.5)	119 (47.6)	190 (27.9)	42 (62.7)
Network providers, *n*		18	11	3	1
Frequency band^c^, *n* (%)					
Band 48	TDD 3550–3700 MHz	—	39 (15.6)	—	12 (17.9)
Band 77	TDD 3300–4200 MHz	—	61 (24.4)	—	0 (0.0)
Band 7	DL 2620–2690 MHz	727 (25.1)	0 (0.0)	225 (33.1)	—
Band 38	TDD 2570–2620 MHz	18 (0.6)	0 (0.0)	0 (0.0)	—
Band 1	DL 2110–2170 MHz	538 (18.6)	38 (15.2)	173 (25.4)	21 (31.3)
Band 3	DL 1805–1880 MHz	1113 (38.4)	75 (30.0)	241 (35.4)	0 (0.0)
Band 8	DL 925–960 MHz	31 (1.1)	9 (3.6)	5 (0.7)	0 (0.0)
Band 5	DL 869–894 MHz	0 (0.0)	0 (0.0)	27 (4.0)	0 (0.0)
Band 20	DL 791–821 MHz	381 (13.2)	0 (0.0)	0 (0.0)	34 (50.7)
Band 28	DL 758–803 MHz	89 (3.1)	14 (5.6)	9 (1.3)	0 (0.0)
Band 14	DL 758–768 MHz	0 (0.0)	14 (5.6)	0 (0.0)	0 (0.0)

*Note:* A dash (–) indicates that the value is not applicable.

Abbreviations: DL = downlink, LTE = Long Term Evolution, MHz = Megahertz, NR = New Radio, TDD = time division duplexing.

^a^ 876 (30.2%) were recorded in Switzerland, 762 (26.3%) in the Netherlands, 504 (17.4%) in Spain, 410 (14.2%) in Greece, 297 (10.3%) in France, and 48 (1.7%) in Germany.

^b^ 83 (33.2%) were recorded in Greece, 78 (31.2%) in the Netherlands, 68 (27.2%) in Spain, and 21 (8.4%) in Switzerland.

^c^ Frequency bands reported using the standardised 3GPP band numbering system.

Serving‐cell signal strength was consistently higher in Australia than in Europe (Table 2, Figure S2), with RSRQ values also indicating somewhat better network quality (Table 2, Figure S3). Environmental RF‐EMF exposure in the serving band was also higher in Australia than in Europe (Table 2). LTE‐RSRP and LTE‐RSSI were strongly correlated (Europe: Spearman *ρ* = 0.98; Figure S4). In contrast, LTE‐RSRQ showed only moderate correlation with LTE‐RSRP (Europe: *ρ* = 0.39; Australia: *ρ* = 0.35; Figure S4–S5) and weak correlation with LTE‐RSSI (Europe: *ρ* = 0.16; Figure S4). NR‐ssRSRQ was moderately correlated with NR‐ssRSRP (Europe: *ρ* = 0.24; Australia: *ρ* = 0.47; Figure S4–S5).

**Table 2:** Summary of signal strength, signal quality, and serving‐band exposure for LTE and NR serving‐cell intervals in Europe and Australia.

	Europe	Australia
*Signal strength indicators*		
LTE‐RSRP (dBm)		
Mean (SD)	−94.1 (14.7)	−80.1 (12.9)
Median (Q1, Q3)	−93.7 (−104.1, −83.2)	−78.2 (−88.9, −70.8)
Min. ‐ Max.	−138.3, −52.0	−118.9, −49.6
N	2897	680
LTE‐RSSI (dBm)		
Mean (SD)	−83.3 (13.6)	—
Median (Q1, Q3)	−82.7 (−92.0, −73.8)	—
Min. ‐ Max.	−120.1, −49.4	—
N	2481	0
NR‐ssRSRP (dBm)		
Mean (SD)	−92.0 (12.8)	−74.7 (11.8)
Median (Q1, Q3)	−92.6 (−102.4, −82.6)	−75.3 (−81.9, −67.7)
Min. ‐ Max.	−119.4, −65.3	−115.3, −45.2
N	250	67
*Signal quality indicators*		
LTE‐RSRQ (dB)		
Mean (SD)	−11.3 (3.0)	−9.7 (2.7)
Median (Q1, Q3)	−11.3 (−13.3, −9.1)	−9.4 (−11.3, −7.7)
Min. ‐ Max.	−20.0, −3.0	−21.0, −4.1
N	2897	680
NR‐ssRSRQ (dB)		
Mean (SD)	−12.0 (2.9)	−11.6 (1.5)
Median (Q1, Q3)	−11.7 (−13.0, −11.0)	−11.2 (−12.5, −10.2)
Min. ‐ Max.	−25.8, −4.9	−16.8, −9.9
N	250	67
*Environmental RF‐EMF exposure*		
In LTE serving band (dBµVm^−1^)		
Mean (SD)	108.6 (12.2)	116.6 (9.6)
Median (Q1, Q3)	97.1 (90.8, 106.2)	110.3 (102.8, 116.2)
Min. ‐ Max.	65.6, 125.5	74.5, 131.0
N	2897	680
In NR serving band (dBµVm^−1^)		
Mean (SD)	110.1 (11.3)	120.0 (8.8)
Median (Q1, Q3)	102.7 (93.5, 109.6)	112.0 (104.9, 116.9)
Min. ‐ Max.	69.7, 123.7	90.8, 132.0
N	250	67

Abbreviations: dBm = decibel‐milliwatts, LTE = Long Term Evolution, NR = New Radio, Q1 = first quartile, Q3 = third quartile, RSRP = reference signal received power, RSSI = received signal strength indicator, RSRQ = reference signal received quality, SD = standard deviation.

At the location level, environmental RF‐EMF exposure from LTE networks was higher in Australia than in Europe. Mean environmental RF‐EMF exposure from LTE networks was 114.9 ± 10.3 dBµVm^−1^ (0.56 ± 0.43 Vm^−1^) across 61 locations in Europe and 123.3 ± 8.3 dBµVm^−1^ (1.47 ± 0.97 Vm^−1^) across 48 locations in Australia (Table S16). Differences in average environmental RF‐EMF exposure between Europe and Australia may reflect differences in sampling locations, as more than half of Australian measurements were conducted under LoS conditions, while indoor settings were more frequent in Europe (Tables S1–S7). In Europe, 5G‐NR signal indicators were available at 41 of the 61 spot measurement locations, indicating detectable 5G‐NR coverage at these sites. Mean environmental exposure from LTE and NR networks across these locations was 115.8 ± 7.6 dBμVm^−1^ (0.62 ± 0.40 Vm^−1^; Table S16).

### 3.1 Technical Conversion Functions

LTE signal strength and signal quality indicators each improved model fit, as removing either predictor resulted in a significantly worse fit (LRT *p* < 0.001 for both comparisons). Signal strength indicators (LTE‐RSRP or LTE‐RSSI) showed a positive association with environmental exposure, whereas signal quality (LTE‐RSRQ) was inversely associated (Table 3, Figure 1). In Europe, LTE‐RSRP and LTE‐RSSI showed very similar associations with environmental exposure, although models based on LTE‐RSRP performed marginally better, with slightly lower root mean square errors compared with LTE‐RSSI‐based models (7.47 vs 7.61 dBµVm^−1^; Table 3). In addition, the inverse association between signal quality and environmental exposure (i.e., negative coefficient estimates for LTE‐RSRQ; Table 3) was more pronounced when RSRQ was modelled alongside RSRP than alongside RSSI.

**Table 3:** Association estimates^a^ between signal indicators and environmental RF‐EMF exposure, stratified by region and mobile network technology.

Model	Intercept *β*_0_ (95% CI)	Signal strength *β*_1_ (95% CI)	Signal quality *β*_2_ (95% CI)	Adjusted *R* ^2^	RMSE
Europe					
Technical functions					
LTE‐RSRP + LTE‐RSRQ (*n* = 2897)	151.80 (150.01, 153.59)	0.74 (0.72, 0.76)	−1.33 (−1.44, −1.23)	0.63	7.47
LTE‐RSSI + LTE‐RSRQ (*n* = 2481)	151.00 (148.99, 153.01)	0.72 (0.70, 0.74)	−0.56 (−0.67, −0.46)	0.61	7.61
NR‐ssRSRP (*n* = 250)	152.82 (144.93, 160.71)	0.56 (0.48, 0.65)	—	0.40	8.65
Map functions					
LTE‐RSRP + LTE‐RSRQ (*n* = 61)	161.47 (155.04, 167.90)	0.86 (0.78, 0.94)	−1.28 (−1.77, −0.79)	0.89	3.29
LTE‐RSSI + LTE‐RSRQ (*n* = 61)	162.46 (156.42, 168.51)	0.86 (0.79, 0.94)	−0.50 (−0.93, −0.07)	0.91	3.07
NR‐ssRSRP (*n* = 48)	134.34 (122.00, 146.68)	0.27 (0.13, 0.41)	—	0.23	6.53
Combined received power^b^ (*n* = 61)	154.52 (146.73, 162.30)	0.56 (0.46, 0.66)	—	0.69	4.67
Australia					
Technical functions					
LTE‐RSRP + LTE‐RSRQ (*n* = 680)	146.69 (143.66, 149.72)	0.62 (0.58, 0.66)	−1.23 (−1.43, −1.04)	0.57	6.29
NR‐ssRSRP (*n* = 67)	142.55 (130.98, 154.12)	0.41 (0.26, 0.56)	—	0.29	7.24
Map functions					
LTE‐RSRP + LTE‐RSRQ (*n* = 48)	167.02 (161.17, 172.88)	0.82 (0.74, 0.90)	−0.64 (−1.05, −0.22)	0.90	2.53

*Note:* Regression coefficients (β) are reported with 95% confidence intervals. A dash (–) indicates that no signal quality indicator was included in the model (LRT *p* > 0.05).

Abbreviations: LTE = Long Term Evolution, NR = New Radio, RF‐EMF = radiofrequency electromagnetic fields, RSRP = reference signal received power, RSSI = received signal strength indicator, RSRQ = reference signal received quality, RMSE = root mean square error.

^a^ Estimates from log‐linear regression models relating RF‐EMF environmental exposure (dBµV m^−1^) to signal strength (dBm) and signal quality (dB) indicators. Technical functions investigated the association between serving‐cell indicators and environmental exposure in the serving frequency band (unit of analysis: serving‐cell interval aggregates). Map functions investigated the association between location‐level signal indicators to environmental exposure from LTE or LTE + NR networks.

^b^ Combined received power corresponds to the sum of all power contributions (LTE‐RSRP + NR‐ssRSRP) across providers at each location.

**Figure 1:**
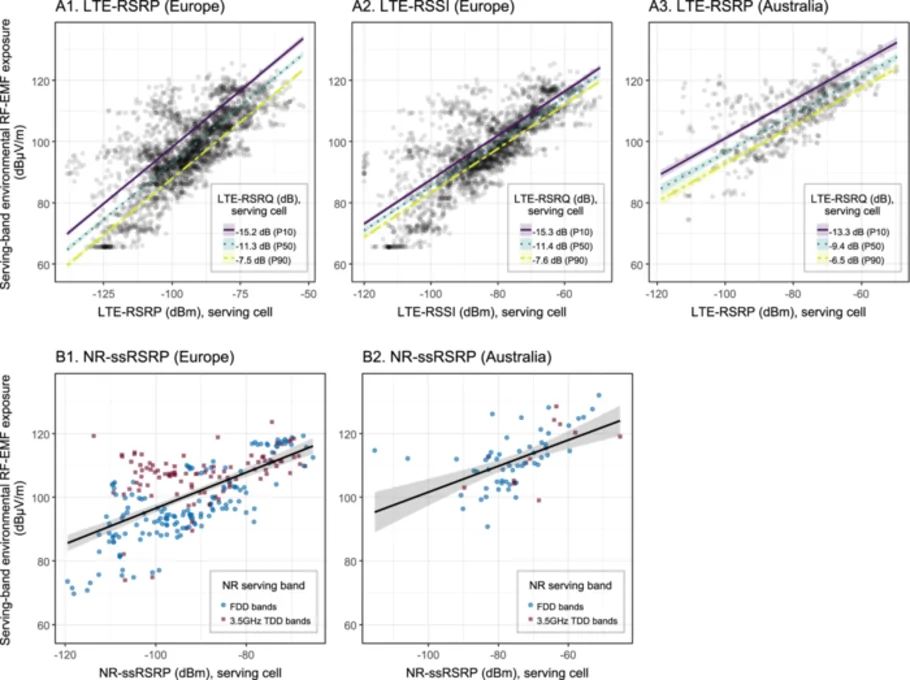
Technical function models showing associations between serving‐cell signal indicators and environmental RF‐EMF exposure in the serving band. Panels A1–A3 depict LTE associations in Europe and Australia, with fitted log‐linear regression lines shown for model predictions at the 10th (P10), 50th (P50), and 90th (P90) percentiles of the observed LTE‐RSRQ distribution. Points represent serving‐cell intervals and shaded areas represent 95% confidence intervals for the fitted regression lines. Panels B1–B2 show NR associations in Europe and Australia; solid lines and shaded areas indicate fitted log‐linear regressions and 95% confidence intervals. Points represent serving‐cell intervals colour and shape‐coded by serving band, shaded areas represent 95% confidence intervals for the fitted regression lines.

For NR, only the signal strength indicator (ssRSRP) explained variation in environmental RF‐EMF exposure (LRT *p* < 0.001), while signal quality (ssRSRQ) did not significantly improve model fit when added to the model in either Europe (LRT *p* = 0.462) or Australia (LRT *p* = 0.099). Thus, signal quality was not included in the final models. Associations between NR‐ssRSRP and environmental exposure were positive but less steep than those observed for LTE signal strength indicators (Table 3, Figure 1). Notably, measured exposure tended to be higher and more variable in the 3.5GHz TDD bands than in FDD bands, particularly at lower ssRSRP values (Figure 1, Panel B1).

Overall patterns were consistent between Europe and Australia, with similar directions and relative effect sizes for LTE signal strength and signal quality indicators, as well as for the NR signal strength indicator (Table 3; Figure 1).

When extending the base technical conversion function (model 0) to include additional network and contextual covariates (model 1), serving‐cell signal strength indicators remained the main contributors to variability in serving‐band environmental exposure (Table 4). Serving‐cell signal strength consistently showed the largest unique contribution to exposure variability, with semi‐partial *R* ^2^ values ranging from 0.27 to 0.29 for LTE and 0.10 for NR in Europe, and from 0.08 to 0.13 in Australia. Signal quality indicators contributed a markedly smaller share of explained variance than signal strength (semi‐partial *R* ^2^ ≤ 0.03 across all LTE models), with larger effect sizes when modelled alongside LTE‐RSRP than when paired with LTE‐RSSI.

**Table 4:** Relative contributions of signal, network, and contextual factors to variability in serving‐band environmental RF‐EMF exposure from technical functions.^a^

	Europe data			Australia data	
	LTE‐RSRP model	LTE‐RSSI model	NR‐ssRSRP model	LTE‐RSRP model	NR‐ssRSRP model
Marginal *R* ^2^	0.691	0.681	0.652	0.697	0.301^b^
ICC (provider)	0.110	0.096	< 0.001	0.016	—
Semi‐partial *R* ^2^					
Serving‐cell signal strength	0.287	0.270	0.101	0.125	0.083
Serving‐cell signal quality	0.030	0.005	—	0.024	—
Serving band	0.006	0.006	0.122	0.030	0.002
Activity	0.001	< 0.001	0.002	< 0.001	0.003
Smartphone model	0.002	0.001	0.003	—	—
Antenna situation	—	—	—	0.050	0.044
Indoor/outdoor setting	0.013	0.012	0.005	—	—
Microenvironment	0.012	0.012	0.034	0.032	0.078
Urbanicity	0.002	0.002	0.008	0.009	0.021
Country	0.036	0.050	< 0.001	—	—

Abbreviations: ICC = intraclass correlation coefficient, LTE = Long Term Evolution, NR = New Radio, RF‐EMF = radiofrequency electromagnetic fields, RSRP = reference signal received power, RSSI = received signal strength indicator, RSRQ = reference signal received quality.

^a^ Values from log‐linear (mixed) regression models (model 1) extending the base technical‐function model (model 0) with additional network and contextual covariates. A dash (–) indicates that the variable was not included in the model.

^b^ Adjusted *R* ^2^ from log‐linear model without random effects (only recordings from one network provider).

Random effects for network provider accounted for a moderate proportion of variance in European LTE models (ICC = 0.10–0.11), a small proportion in Australian LTE (ICC = 0.02), and negligible variance in European NR (Table 4).

Other network and contextual variables contributed comparatively little to the explained variance (Table 4). This reflects their modest incremental explanatory contribution beyond the signal indicators included in the base model, rather than a lack of association with exposure. Serving frequency band contributed moderately to exposure variance in European NR models (semi‐partial *R* ^2^ = 0.12) but was otherwise small in LTE models in both regions (semi‐partial R^2^ ≤ 0.03). Country explained a modest unique proportion of exposure variance in Europe (semi‐partial *R* ^2^ = 0.04‐0.05), while urbanicity and microenvironment each accounted for small unique proportions of exposure variability, with somewhat higher contributions observed for NR compared with LTE. Indoor/outdoor setting contributed marginally to exposure variance in European models (about 0.01), while antenna situation in Australia explained a modest proportion in LTE and NR models (semi‐partial *R* ^2^ = 0.04‐0.05). Lastly, smartphone model and activity consistently explained negligible exposure variance (< 0.01).

### 3.2 Map Conversion Function

Map conversion functions generally showed stronger associations and better model performance than technical functions, explaining a larger proportion of variance (adjusted *R* ^2^ up to 0.91 for LTE models in Europe and Australia, compared with ≤ 0.63 for technical functions) and yielding substantially lower root mean square values (Table 3; Figure 2). Location‐level signal strength in LTE cells (RSRP or RSSI summed across providers) showed a strong positive association with environmental RF‐EMF exposure from LTE networks, whereas average LTE‐RSRQ across providers was inversely associated (Table 3; Figure 2). LTE map functions were comparable between Europe and Australia, with similar magnitudes of association estimates for signal strength and to some extent also for signal quality indicators.

**Figure 2:**
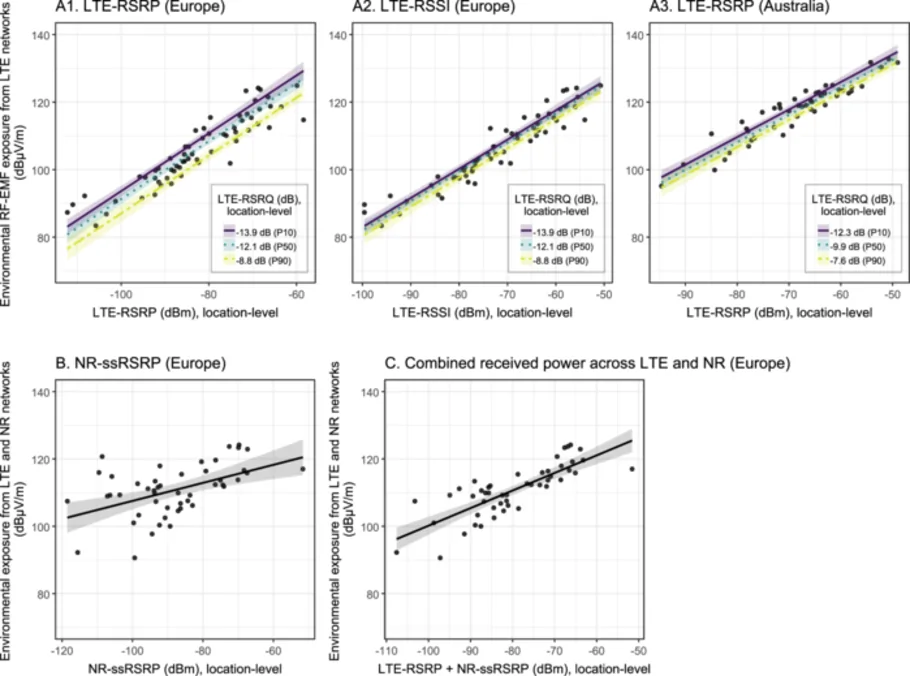
Map function models showing associations between location‐level signal indicators and total environmental RF‐EMF exposure from mobile networks. Panels A1–A3 depict associations between total LTE signal strength (RSRP or RSSI) and total LTE environmental exposure in Europe and Australia, with fitted log‐linear regression lines shown for model predictions at the 10th (P10), 50th (P50), and 90th (P90) percentiles of the observed location‐level LTE‐RSRQ distribution. Panel B shows the association between total NR‐ssRSRP and total mobile environmental exposure in Europe. Panel C shows the association between total received power and total mobile environmental exposure in Europe. Points represent location‐level aggregates and shaded areas represent 95% confidence intervals for the fitted regression lines.

Consistent with observations from NR technical functions, inclusion of signal quality (ssRSRQ) did not significantly improve model fit and was therefore not retained in the final model (LRT *p* = 0.554). Associations between NR‐ssRSRP summed across providers and environmental exposure from both LTE and NR networks were weak (Table 3; Figure 2). In comparison, using the combined received power indicator across LTE and NR technologies as a predictor resulted in an intermediate model performance between NR‐only and LTE‐only models. NR map functions could not be fitted for Australia because NR indicators were recorded for only one network provider, precluding the estimation of aggregated NR power contributions across different providers at the location level.

## 4 Discussion

In this multi‐country measurement study, we derived empirical conversion functions describing the association between smartphone signal indicators and average environmental RF‐EMF exposure from mobile networks. Across six European countries and Australia, signal strength indicators showed a strong, consistent positive association with measured environmental exposure. Signal quality indicators contributed modestly and inversely to LTE models, but not to NR models. These associations were consistent in direction and broadly similar in magnitude across regions and strengthened when measurements from different cells and providers were combined at the location level.

Previous studies using mobile phone‐derived data focused mainly on usage characteristics such as phone position, laterality, and call frequency and duration (Bhatt et al. 2016, 2022; Goedhart et al. 2015; Inyang et al. 2009; Kiyohara et al. 2018; Vrijheid et al. 2009). More recently, smartphone applications like XMobiSensePlus (Mazloum et al. 2020), ElectroSmart (Boussad et al. 2022), and Quanta Monitor (Bhatt et al. 2018) have been used to track network‐related parameters. Some approaches have also been proposed to estimate environmental exposure using signal strength indicators (Boussad et al. 2021; Schießl 2023a; Schießl et al. 2021). In particular, Schießl et al. (2023a, 2023b) derived calibration factors as offsets in dB between smartphone received power and electric field strength. These calibration factors were developed within a framework that establishes a 1:1 proportionality between smartphone signal strength and code‐selective field measurements of maximum field strength (Schießl 2023a, 2023b). In contrast, we modelled the empirical association between smartphone signal indicators (used as proxies for propagation conditions and network variability) and measured average environmental RF‐EMF exposure from mobile networks. These quantities are physically related but not equivalent, because the exposimeter used in this study captured contributions from multiple sources within predefined frequency bands, whereas the smartphone indicators refer to specific reference signals of the serving and neighbouring cells. Under such conditions, deviation of the slope of signal strength from unity is expected and may arise because measured environmental exposure was likely influenced by contributions that are not fully captured by the app indicators. In addition, the fact that our target quantity is mean environmental RF‐EMF exposure rather than maximum field strength, as in Schießl (2023a, 2023b), is also an important distinction and corresponds more directly to the exposure metric relevant for population‐based studies and risk communication, where long‐term exposure averages are of primary interest.

We incorporated signal quality indicators to capture network‐related effects that may influence environmental exposure independently of propagation conditions. For LTE, signal quality showed a consistent inverse association with environmental exposure from mobile networks, indicating higher exposure at lower signal quality for a given received power. This association was more pronounced when RSRQ was modelled alongside RSRP than when paired with RSSI. This is consistent with the underlying definitions of these indicators (3GPP 2025a), and RSSI intrinsically contains part of the information captured by RSRQ. The similar behaviour of LTE‐RSRP and LTE‐RSSI in the conversion functions likely also reflects the relatively limited variation in LTE downlink power under the network conditions captured in this study, which appeared to be predominantly under low‐to‐moderate load.

Model performance for NR functions was lower and less consistent than for LTE, with signal strength (ssRSRP) explaining less variance in environmental exposure and showing markedly weaker association slopes. These weaker associations may reflect differences in how the LTE and NR signal indicators relate to total downlink power. In LTE, RSRP is derived from cell‐specific reference signals that occupy a relatively large and fixed fraction of time‐frequency resources (3GPP 2025a; Aerts et al. 2019), making it a reasonable proxy for average transmitted downlink power and thus for environmental exposure. In NR, ssRSRP measures the received power of the synchronisation signal block (SSB), rather than that of user‐data traffic transmissions (3GPP 2025b). At 3.5GHz, SSBs may provide cell‐wide coverage through a broadcast beam or a set of periodically switched beams, whereas traffic is dynamically beamformed towards scheduled users (Aerts et al. 2019). Consequently, ssRSRP may be detectable at a location even when no traffic beam is directed towards it. Moreover, NR typically operates with larger, dynamically allocated bandwidths; thus, total downlink power can vary considerably at similar signal strength levels, especially under full‐load conditions. The weaker correspondence between NR‐ssRSRP and measured exposure in TDD bands may also partly reflect the imperfect separation of environmental (downlink) and phone related (uplink) components in exposimeter TDD measurements, as well as potential contributions from nearby devices and downlink beams directed to other users. Lastly, in the NR map function, environmental exposure reflected the current overlap between LTE and NR deployments. Under these conditions, exposure may remain dominated by LTE transmissions, consistent with the improved performance of models based on combined received power indicator across LTE and NR technologies, compared with NR‐ssRSRP models. Overall, our findings suggest that NR‐ssRSRP is a less reliable proxy for average environmental RF‐EMF exposure from mobile networks than LTE signal indicators.

Smartphones are not designed as RF‐EMF measurement devices. Consequently, recorded signal indicators are sensitive to device orientation, user interaction, and the direction in which the serving base station transmits power (Boussad et al. 2021). Although antenna directionality effects are reduced under multipath propagation conditions typical of real‐life environments (Boussad et al. 2021; Schießl 2023a), short‐term variability remains present but can be compensated by averaging repeated measurements collected under varying conditions (Schießl 2023a). In our study, unexplained variability in the signal‐exposure relationship appeared to be driven by short‐term network dynamics and small‐scale propagation effects rather than by stable contextual factors. This is supported by the fact that signal strength indicators consistently explained the largest proportion of environmental exposure variance, while contextual covariates added only small unique contributions when extending the base technical models. We also observed a reduction in RMSE when measurements were aggregated at each location, indicating that much of the residual variability in the technical functions operates at short temporal and spatial scales. In addition, the marked improvement in model performance when combining contributions from different cells and providers is consistent with a more complete representation of the relevant sources of environmental exposure. In other words, single measurements are subject to substantial uncertainty, but aggregation of a large number of observations across different network providers yields a more meaningful estimate of environmental RF‐EMF exposure from mobile networks.

From an epidemiological perspective, the accuracy of long‐term average exposure estimates is more relevant than the precision of individual measurement points. Moreover, the structure of measurement error is more important than absolute prediction accuracy. Differential measurement error, which poses the greater threat to epidemiological validity, would require that the signal‐exposure relationship differ systematically across population subgroups in ways correlated with the outcomes under study (Lash et al. 2021). Across the variables examined, we found limited evidence for such potential structure in LTE models. Smartphone model, usage activity, antenna situation, indoor/outdoor setting, microenvironment, and urbanicity each explained only small unique proportions of environmental exposure variance, and network provider effects were moderate at most. This indicates that much of their association with the measured environmental RF‐EMF exposure is shared with, and captured by, the signal indicators included in the model. At the population level, location‐based exposure estimation using the developed LTE map functions would likely be dominated by Berkson‐type error, in which individual exposures vary around an unbiased location mean (Yland et al. 2022). Such non‐differential error would increase residual variance but not bias association estimates, preserving the validity of epidemiological inference. Results for NR models were less consistent. In European NR analyses, the serving frequency band explained as much unique variance in environmental exposure as the signal strength indicator itself. Because it is unclear whether the NR signal strength indicator is a good proxy for exposure and because the number of observations for NR analyses was limited, conclusions regarding the measurement‐error structure for NR should be considered preliminary.

Beyond epidemiological applications, our findings are also relevant for risk communication, where transparency and interpretability are often more important than precise point estimates. Exposure estimates based on signal indicators offer an intuitive and scalable way to communicate relative exposure differences across locations. Although estimation is imperfect, the exposure contrasts observed between locations are sufficiently large to support meaningful communication of spatial patterns. Within the ETAIN framework, future work will extend this approach beyond environmental exposure by linking smartphone signal indicators to mobile phone output power, for estimation of exposure from mobile phone use alongside environmental exposure from mobile networks. In addition, an online tool developed within the ETAIN project (the EMF dose calculator (ETAIN 2026)) is designed to inform the general public about daily RF‐EMF dose by integrating contributions from both near‐field and far‐field sources.

Key strengths of this study include the use of measurements across different countries, network providers, microenvironments, and urbanicity levels. We focused on deriving conversion functions describing the association between smartphone signal indicators and average environmental exposure from mobile networks under typical use conditions, which is well aligned with the needs of epidemiological research and risk communication. Additionally, the explicit decomposition of exposure variability using mixed‐effects models allowed identification of the dominant contributors to variability and provided insight into potential sources of systematic bias.

Several limitations should be acknowledged. Technology use and frequency band allocation could not be controlled, resulting in few observations for some frequency bands and precluding connection to available 5G‐SA networks in Australia and Germany. In addition, many NR TDD observations during active phone use were excluded because measurements may reflect contributions from the phone's uplink transmission. As a result, NR analyses were underpowered and may not fully represent the range of exposure variability. Another limitation is that the ExpoM‐RF4 samples each frequency band during short measurement windows, corresponding to only a small fraction of elapsed time for any given band. Although repeated samples were averaged over serving‐cell intervals or measurement locations, this assumes that the sampled windows are representative of the exposure distribution during the aggregation period. Deviations from this assumption may contribute to residual variability in the conversion functions. Recordings were restricted to Android smartphones running a recent version of the operating system. Although signal strength indicators can vary by system‐on‐a‐chip and Android version (Laplanche et al. 2025), we were unable to assess whether such device‐specific factors contributed to additional variance in the technical conversion functions. In addition, the distributions of signal quality indicators (Figure S4), particularly ssRSRQ, suggest possible quantisation or capping of the recorded values, indicating that these Android‐reported indicators may behave differently from their formal definitions. This may partly explain why signal quality indicators contributed only modestly to LTE models and did not improve NR models. We included a broad range of activities to characterise typical phone usage across diverse propagation conditions and network environments, including locations where higher network load was expected (e.g., city centres). However, the observed strong correlation between LTE‐RSRP and LTE‐RSSI may indicate that measurements were still predominantly conducted under low‐to‐moderate network load conditions. Future studies could aim to capture higher‐load scenarios more systematically, for example by combining measurements in crowded environments with high‐traffic phone activities such as file downloads or streaming. This would help better characterise exposure under high‐traffic conditions and assess the transportability of the derived conversion functions to other settings and network configurations. Lastly, the capped dynamic range of smartphone signal indicators limits the characterisation of very high or very low field strengths. However, this constraint would be less critical for epidemiological research and risk communication, which primarily rely on relative exposure contrasts rather than absolute values.

## 5 Conclusions

Signal indicators collected with the ETAIN 5G Scientist app provide a promising basis for characterising average environmental RF‐EMF exposure from mobile networks at the population level. LTE signal indicators were found to be more informative proxies for environmental exposure, whereas NR signal indicators currently appear to be less reliable. While smartphones are not measurement instruments and cannot replace dedicated devices for compliance assessment, they may offer a scalable, low‐cost approach to RF‐EMF exposure assessment that is well aligned with the needs of epidemiological research and risk communication. However, future studies should assess the validity of this approach in independent settings and populations.

## Author Contributions

**Nekane Sandoval Diez** contributed to investigation, methodology, conceptualisation, data collection, data curation, formal analysis, visualisation, writing – original draft. **Lea Belácková** contributed to methodology, data collection, writing – review and editing. **Hamed Jalilian** contributed to methodology, formal analysis, writing – review and editing. **Adriana Fernandes Veludo** contributed to methodology, formal analysis, writing – review and editing. **Lloyd Roga** contributed to methodology, validation, writing – review and editing. **Isabelle Deltour** contributed to methodology, data collection, writing – review and editing. **Stuart Henderson** contributed to methodology, data collection, writing – review and editing. **Sarah Loughran** contributed to methodology, data collection, supervision, writing – review and editing. **Arno Thielens** contributed to conceptualisation, investigation, methodology, validation, writing – review and editing. **Marco Zahner** contributed to conceptualisation, investigation, methodology, validation, software development, writing – review and editing. **Jürg Fröhlich** contributed to conceptualisation, investigation, methodology, validation, software development, funding acquisition, validation, writing – review and editing. **Anke Huss** contributed to conceptualisation, investigation, methodology, project administration, funding acquisition, validation, and writing – review and editing. **Martin Röösli** contributed to conceptualisation, investigation, methodology, supervision, funding acquisition, validation, formal analysis, and writing – review and editing. All authors have read and approved the final manuscript.

## Conflicts of Interest

The authors declare no conflicts of interest.

## Supporting information


Supporting File

## Data Availability

The data and analytical code supporting the findings of this study are openly available through the Yoda Data Publication Platform of Utrecht University at: https://doi.org/10.24416/UU01-08PZ98.
