# Supplementary material for: Estimating Environmental RF‐EMF Exposure From Mobile Networks Using Signal Indicators Collected With the ETAIN 5G Scientist App
Source: Bioelectromagnetics. 2026 Oct 13;47(7):e70071. doi: 10.1002/bem.70071 (PMC13659925; doi:10.1002/bem.70071)
Supplement: Supplementary file 1 [file BEM-47-0-s001.pdf]

Supplementary Material

Estimating environmental RF-EMF exposure from mobile networks using signal indicators collected with the ETAIN 5G Scientist app

Nekane Sandoval-Diez[1,2], Lea Beláckova[3], Hamed Jalilian[1,2], Adriana Fernandes Veludo[1,2], Lloyd Roga[3], Isabelle Deltour[4], Stuart Henderson[5], Sarah Loughran[5], Arno Thielens[6], Marco Zahner[7], Jürg Fröhlich[7], Anke Huss[3], and Martin Röösli[1,2,*]

[1] Swiss Tropical and Public Health Institute, Allschwil, Basel-Land, Switzerland

[2] University of Basel, Basel, Switzerland

[3] Institute for Risk Assessment Sciences, Utrecht University, Utrecht, The Netherlands

[4] International Agency for Research on Cancer, Lyon, France

[5] Australian Radiation Protection and Nuclear Safety Agency, Yallambie, Victoria, Australia

[6] ASRC, The Graduate Center of the City University of New York, New York City, USA

[7] Fields at Work LLC, Zürich, Switzerland

*Corresponding author: martin.roosli@swisstph.ch

Table of contents: Tables

Table S1. Selected measurement locations in France (n=4) 4
Table S2. Selected measurement locations in the Netherlands (n=14) 4
Table S3. Selected measurement locations in Switzerland (n=15)...................................... 4
Table S4. Selected measurement locations in Spain (n=14) 5
Table S5. Selected measurement locations in Greece (n=14) 5
Table S6. Selected measurement locations in Germany (n=15) 6
Table S7. Selected measurement locations in Australia (n=55).. 6
Table S8. Antenna line-of-sight (LoS) conditions for each measurement location and network provider in Australia.. 7
Table S9. ExpoM-RF4 frequency bands (n=35) programmed for measurements in Europe 9
Table S10. ExpoM-RF4 frequency bands (n=33) programmed for measurements in Australia 10
Table S11. Smartphone models and network providers used in each country.................... 11
Table S12. Activities performed with the phone during measurements in each country...... 11
Table S13. ExpoM-RF4 band pairs considered for crosstalk correction* (see Section 2.4.1). 12
Table S14. Observations for NR serving-cell intervals reported by the ETAIN 5G Scientist app (raw data recorded every second) by activity, serving frequency band, and country........... 13
Table S15. Locations excluded from map conversion function analyses. 14
Table S16. Summary of signal strength and environmental exposure for location-level aggregates in Europe and Australia... 15

Table of contents: Boxes

Box S1. Conversion of signal strength or quality indicators to linear scale......................... 16
Box S2. Conversion of electric field strength values to power flux density......................... 16
Box S3. Computation of mean signal strength and quality indicators for technical conversion functions. 17
Box S4. Computation of mean environmental RF-EMF exposure in the serving band for technical conversion functions... 17
Box S5. Computation of location-level signal indicator aggregates for map conversion functions. 18
Box S6.Computation of mean environmental RF-EMF exposure from mobile networks at each location for map conversion functions... 20
Box S7. Conversion of electric field strength to dBµV·m^{-1}... 21

Table of contents: Figures

Figure S1. Cumulative distribution functions of LTE-RSRP and LTE-RSSI recorded with the ETAIN 5G Scientist app by smartphone model (raw data recorded every second) for Europe and Australia (data across all countries). 22
Figure S 2. Scatterplots between location-level NR-ssRSRP indicators and measured environmental RF-EMF exposure from LTE and NR networks. 23
Figure S3. Cumulative distribution functions of serving-cell LTE and NR signal strength indicators in Europe and Australia (serving-cell interval aggregates). 24
Figure S4. Cumulative distribution functions of serving-cell LTE and NR signal quality indicators in Europe and Australia (serving-cell interval aggregates) 25
Figure S 5. Correlation between signal strength indicators and signal quality indicators collected with the ETAIN 5G Scientist app (data from Europe). 26
Figure S6. Correlation between signal strength indicators and signal quality indicators collected with the ETAIN 5G Scientist app (data from Australia). 27

Table S1. Selected measurement locations in France (n=4)

**City/Town (n),** ***urbanicity***	**Location (n)**	**Microenvironment**	**Setting**
Lyon (4), *Medium-sized city*	Place Bellecour (1)	Centre or residential	Outdoor
	Gerland district (1)	Commercial or industrial	Outdoor
	Gare de Lyon Part-Dieu (1)	Transportation station	Indoor
	Parc de la Tête d'Or (1)	Green area	Outdoor

Table S2. Selected measurement locations in the Netherlands (n=14)

**City/Town (n),** ***urbanicity***	**Location (n)**	**Microenvironment**	**Setting**
Amsterdam (5), *Big city*	Leidseplein (1)	Centre or residential	Outdoor
	Amsteldijk (1)	Centre or residential	Outdoor
	Amsterdam Zuid (1)	Commercial or industrial	Outdoor
	Amsterdam Central Station (1)	Transportation station	Indoor
	Vondel Park (1)	Green area	Indoor
Utrecht (5), *Medium-sized city*	Mariaplaats (1)	Centre or residential	Outdoor
	Kanaleneiland (1)	Centre or residential	Indoor
	Science Park (1)	Commercial or industrial	Indoor
	Utrecht Central Station (1)	Transportation station	Indoor
	Grift Park (1)	Green area	Outdoor
Werkhoven (2), *Town*	Weerdenhof (1)	Centre or residential	Outdoor
	Beverweertseweg (1)	Centre or residential	Outdoor
Zegveld (2), *Town*	Burgemeester Brediusstraat (1)	Centre or residential	Outdoor
	Lindenlaan (1)	Centre or residential	Outdoor

Table S3. Selected measurement locations in Switzerland (n=15)

**City/Town (n),** ***urbanicity***	**Location (n)**	**Microenvironment**	**Setting**
Zurich (6), *Big city*	Polyterrasse (1)	Centre or residential	Outdoor
	ETH building (1)	Centre or residential	Indoor
	Hegibachstrasse (1)	Centre or residential	Indoor
	Sihlcity (1)	Commercial or industrial	Outdoor
	Zurich train station (1)	Transportation station	Indoor
	Rentenwiese (1)	Green area	Outdoor
Basel (7), *Medium-sized city*	Markplatz (1)	Centre or residential	Outdoor
	Bettenstrasse (2)	Centre or residential	Indoor
	Kreuzstrasse (2)	Commercial or industrial	Indoor
	Basel train station (1)	Transportation station	Indoor
	Schützenmattpark (1)	Green area	Outdoor
Gempen (1), *Town*	Gempen Dorf (1)	Centre or residential	Outdoor
Bubendorf (1), *Town*	Gartenstrasse (1)	Centre or residential	Indoor

Table S4. Selected measurement locations in Spain (n=14)

**City/Town (n), *urbanicity***	**Location (n)**	**Microenvironment**	**Setting**
Barcelona (6), *Big city*	Plaza Catalunya (1)	Centre or residential	Outdoor
	Ronda de la Univ. (1)	Centre or residential	Indoor
	Plaza de Sarrià (1)	Centre or residential	Outdoor
	Plaza de Can Fabra (1)	Commercial or industrial	Outdoor
	Estación de Sants (1)	Transportation station	Indoor
	Parc Ciutadella (1)	Green area	Outdoor
Girona (5), *Medium-sized city*	Plaza Catalunya (1)	Centre or residential	Outdoor
	Barri Vell (1)	Centre or residential	Indoor
	Domeney (1)	Commercial or industrial	Outdoor
	Girona train station (1)	Transportation station	Outdoor
	Devesa (1)	Green area	Outdoor
Sant Celoni (2), *Town*	Plaza de la Vila (1)	Centre or residential	Outdoor
	Sant Celoni train station (1)	Transportation station	Outdoor
Besalú (1), *Town*	Plaza del Prat de Sant Pere (1)	Centre or residential	Outdoor

Table S5. Selected measurement locations in Greece (n=14)

**City/Town (n), *urbanicity***	**Location (n)**	**Microenvironment**	**Setting**
Athens (6), *Big city*	Evripidou (1)	Centre or residential	Outdoor
	Koukaki (1)	Centre or residential	Indoor
	Agricultural University of Athens (2)	Commercial or industrial	Indoor and outdoor
	Larissa Station (1)	Transportation station	Indoor
	National Gardens (1)	Green area	Outdoor
Chalcis (5), *Medium-sized city*	Pl. Agiou Nikolaou (1)	Centre or residential	Outdoor
	Agios Minas (1)	Centre or residential	Outdoor
	Immortal Square (1)	Commercial or industrial	Outdoor
	Chalkida Station (1)	Transportation station	Indoor
	New Boúrkou Park (1)	Green area	Outdoor
Aegina (2), *Town*	Aegina port (1)	Centre or residential	Outdoor
	Agia Marina (1)	Centre or residential	Outdoor
Skaramagas (1), *Town*	Agiou Antoniou (1)	Centre or residential	Outdoor

Table S6. Selected measurement locations in Germany (n=15)

**City/Town (n), *urbanicity***	**Location (n)**	**Microenvironment**	**Setting**
Freiburg im Breisgau (15), *Smaller city*	Münsterplatz (3)	Centre or residential	Outdoor
	Vauban (3)	Centre or residential	Outdoor
	Industriegebiet Nord (3)	Commercial or industrial	Outdoor
	Hauptbahnhof (3)	Transportation station	Outdoor
	Seepark (3)	Green area	Outdoor

Table S7. Selected measurement locations in Australia (n=55)

**City/Town (n), *urbanicity***	**Location (n)**	**Microenvironment**	**Setting**
Melbourne (11), *Big city*	Federation Square (1)	Centre or residential	Outdoor
	Exhibition Street (1)	Centre or residential	Outdoor
	Hawthorne (3)	Centre or residential	Outdoor
	Chadstone shopping (3)	Commercial or industrial	Outdoor
	Princes park (3)	Green area	Outdoor
Sydney (13), *Big city*	Opera House (4)	Centre or residential	Outdoor
	Parramatta (3)	Centre or residential	Outdoor
	Bondi Junction (3)	Commercial or industrial	Outdoor
	Central Station (1)	Transportation station	Outdoor
	Centennial park (2)	Green area	Outdoor
Wollongong (8) *Medium-sized city*	Corrimal street (3)	Centre or residential	Outdoor
	Towradgi (1)	Centre or residential	Outdoor
	Wollongong Central (3)	Commercial or industrial	Outdoor
	Wollongong train station (1)	Transportation station	Outdoor
Cairns (11), *Medium-sized city*	Abbott street (3)	Centre or residential	Outdoor
	Manuda (3)	Centre or residential	Outdoor
	Earlville shopping (3)	Commercial or industrial	Outdoor
	Cairns train station (1)	Transportation station	Indoor
	Botanic Gardens (1)	Green area	Outdoor
Kyneton (7), *Town*	High street (3)	Centre or residential	Outdoor
	Baynton Street (1)	Centre or residential	Outdoor
	Mineral Springs reserve (3)	Green area	Outdoor
Gisborne (5), *Town*	New Gisborne (3)	Centre or residential	Outdoor
	Mt. Gisborne (2)	Green area	Outdoor

Table S8. Antenna line-of-sight (LoS) conditions for each measurement location and network provider in Australia

City/Town	Location	ID	Antenna situation*		
			**Vodafone**	**Telstra**	**Optus**
Melbourne	Federation Square	AUS-ML011	LoS close	LoS close	LoS close
Melbourne	Exhibition Street	AUS-ML041	LoS close	LoS close	LoS close
Melbourne	Hawthorne A	AUS-ML021	LoS close	LoS close	LoS close
Melbourne	Hawthorne B	AUS-ML022	LoS far	LoS far	LoS far
Melbourne	Hawthorne C	AUS-ML023	NLoS	NLoS	NLoS
Melbourne	Chadstone shopping A	AUS-ML031	LoS close	LoS close	LoS close
Melbourne	Chadstone shopping B	AUS-ML032	LoS far	LoS far	LoS far
Melbourne	Chadstone shopping C	AUS-ML033	NLoS	NLoS	NLoS
Melbourne	Princes park	AUS-ML051	LoS close	LoS far	LoS close
Melbourne	Princes park	AUS-ML052	LoS far	LoS close	LoS far
Melbourne	Princes park	AUS-ML053	NLoS	NLoS	NLoS
Sydney	Opera House A	AUS-SD010	LoS close	LoS close	LoS close
Sydney	Opera House B	AUS-SD011	LoS close	LoS close	LoS close
Sydney	Opera House C	AUS-SD012	LoS far	LoS far	LoS far
Sydney	Opera House D	AUS-SD013	NLoS	NLoS	NLoS
Sydney	Parramatta A	AUS-SD021	LoS close	LoS close	LoS close
Sydney	Parramatta B	AUS-SD022	LoS far	LoS far	LoS far
Sydney	Parramatta C	AUS-SD023	NLoS	NLoS	NLoS
Sydney	Bondi Junction A	AUS-SD031	LoS close	LoS close	LoS close
Sydney	Bondi Junction B	AUS-SD032	LoS far	LoS far	LoS far
Sydney	Bondi Junction C	AUS-SD033	NLoS	NLoS	NLoS
Sydney	Central Station	AUS-SD041	LoS close	LoS close	LoS close
Sydney	Centennial park A	AUS-SD051	LoS close	LoS close	LoS close
Sydney	Centennial park B	AUS-SD052	NLoS	NLoS	NLoS
Wollongong	Corrimal street A	AUS-WG011	LoS close	LoS close	LoS close
Wollongong	Corrimal street B	AUS-WG012	LoS far	LoS far	LoS far
Wollongong	Corrimal street C	AUS-WG013	NLoS	NLoS	NLoS
Wollongong	Towradgi	AUS-WG023	NLoS	NLoS	NLoS
Wollongong	Wollongong Central A	AUS-WG031	LoS close	NLOS	LOS close
Wollongong	Wollongong Central B	AUS-WG032	LoS far	LoS close	LoS far
Wollongong	Wollongong Central C	AUS-WG033	NLoS	NLoS	NLoS
Wollongong	Wollongong train station	AUS-WG043	NLoS	NLoS	NLoS
Cairns	Abbott street A	AUS-CR011	LoS close	LoS close	LoS close
Cairns	Abbott street B	AUS-CR012	LoS far	LoS far	LoS far
Cairns	Abbott street C	AUS-CR013	NLoS	NLoS	NLoS
Cairns	Manuda A	AUS-CR021	LoS far	LoS close	LoS far
Cairns	Manuda B	AUS-CR022	LoS close	NLOS	LoS close
Cairns	Manuda C	AUS-CR023	NLoS	NLoS	NLoS
Cairns	Earlville shopping A	AUS-CR031	LoS close	LoS close	LoS close
Cairns	Earlville shopping B	AUS-CR032	LoS far	LoS far	LoS far
Cairns	Earlville shopping C	AUS-CR033	NLoS	NLoS	NLoS

Cairns	Cairns train station	AUS-CR043	NLoS	NLoS	NLoS
Cairns	Botanic Gardens	AUS-CR053	NLoS	NLoS	NLoS
Kyneton	High street A	AUS-KY011	LoS close	LoS close	NLoS
Kyneton	High street B	AUS-KY012	LoS far	LoS far	NLoS
Kyneton	High street C	AUS-KY013	NLoS	NLoS	NLoS
Kyneton	Baynton Street	AUS-KY010	NLoS	LoS close	NLoS
Kyneton	Mineral Springs reserve A	AUS-KY021	LoS close	LoS close	LoS close
Kyneton	Mineral Springs reserve B	AUS-KY022	LoS far	LoS far	LoS far
Kyneton	Mineral Springs reserve C	AUS-KY023	NLoS	NLoS	NLoS
Gisborne	New Gisborne A	AUS-GS011	NLoS	LoS close	LoS close
Gisborne	New Gisborne B	AUS-GS012	NLoS	LoS far	LoS far
Gisborne	New Gisborne C	AUS-GS013	NLoS	NLoS	NLoS
Gisborne	Mt. Gisborne A	AUS-GS021	LoS close	LoS close	LoS close
Gisborne	Mt. Gisborne B	AUS-GS022	LoS far	LoS far	LoS far

*LoS close refers to an estimated transmitter distance of approximately 50-100 m, while LoS far corresponds to approximately 150-200 m. NLOS indicates non-line-of-sight conditions.

Table S9. ExpoM-RF4 frequency bands (n=35) programmed for measurements in Europe

**Service Category**	**Band name**	**Centre frequency (MHz)**	**Bandwidth (MHz)**
Broadcast / Infrastructure	FM Radio	97.75	35
	DAB/DAB+	202	75
	Polycom/TETRAPOL	385	35
	TETRAPOL, ISM 433	422.5	35
	PMR/PAMR (digital radio)	452.5	35
	Broadcasting (1)	507.5	75
	Broadcasting (2)	583.5	75
	Broadcasting (3)	659.5	75
	DECT	1897.5	35
Mobile Downlink	**700 FDD DL**	**770.5**	**35**
	**700 FDD SDL**	**748**	**35**
	**800 FDD DL**	**808.5**	**35**
	**900 FDD DL**	**942.5**	**35**
	**1400 FDD DL**	**1479.5**	**75**
	**1800 FDD DL**	**1842.5**	**75**
	**2100 FDD DL**	**2145**	**75**
	**2600 FDD DL**	**2657**	**75**
Mobile Uplink	700 FDD UL	718	35
	800 FDD UL	847	35
	900 FDD UL	897.5	35
	1800 FDD UL	1747.5	75
	2100 FDD UL	1957	75
	2600 FDD UL	2535	75
Mobile TDD	**2600 TDD**	**2592.5**	**35**
	**3500 TDD (1)***	**3475**	**100**
	**3500 TDD (2)***	**3605**	**100**
	**3500 TDD (3)***	**3735**	**100**
WiFi	ISM 2.4 GHz	2438	100
	WiFi 5 GHz (1)	5200	100
	WiFi 5 GHz (2)	5325	100
	WiFi 5 GHz (3)	5450	100
	WiFi 5 GHz (4)	5575	100
	WiFi 5 GHz (5)	5700	100
	WiFi 5.8 GHz (1)	5825	100
	WiFi 5.8 GHz (2)	5950	100

Bands highlighted in **bold red** were included in the analyses; all other bands were programmed on the ExpoM-RF4 for measurement but were not used in the results presented in this paper.

*Bands programmed in the second ExpoM-RF4 device used for measurements in Germany.

DAB/DAB+ = Digital Audio Broadcasting / Digital Audio Broadcasting Plus; DECT = Digital Enhanced Cordless Telecommunications; DL = Downlink; SDL = Supplementary Downlink; FM = Frequency Modulation; FDD = Frequency Division Duplexing; ISM = Industrial, Scientific and Medical band; PMR = Private Mobile Radio; PAMR = Public Access Mobile Radio; TDD = Time Division Duplex; UL = Uplink; WiFi = Wireless Fidelity

Table S10. ExpoM-RF4 frequency bands (n=33) programmed for measurements in Australia

Service Category	Band name	Centre frequency (MHz)	Bandwidth (MHz)
Broadcast / Infrastructure	FM Radio	97.75	35
	DVB-T & DAB	211.5	75
	UHF Paging	411.5	35
	UHF Paging	485	75
	UHF DVB-T	554	75
	UHF DVB-T	638	100
	DECT	1897.5	35
	VHF paging	156.5	35
Mobile Downlink	**700 FDD DL**	**787**	**75**
	**800 FDD DL**	**872.5**	**35**
	**900 FDD DL**	**950**	**35**
	**1800 FDD DL**	**1842.5**	**75**
	**2100 FDD DL**	**2140**	**75**
	**2600 FDD DL**	**2655**	**75**
Mobile Uplink	700 FDD UL	720	75
	800 FDD UL	835	35
	900 FDD UL	902.5	35
	1800 FDD UL	1747.5	75
	2100 FDD UL	1950	75
	2600 FDD UL	2535	75
Mobile TDD	**2300 TDD**	**2350**	**100**
	**3500 TDD (1)***	**3462.5**	**75**
	**3500 TDD (2)***	**3550**	**100**
	**3500 TDD (3)***	**3650**	**100**
	**3500 TDD (4)***	**3750**	**100**
WiFi	ISM 2.4 GHz	2450	100
	Wi-Fi 5 GHz (1)	5200	100
	Wi-Fi 5 GHz (2)	5300	100
	Wi-Fi 5 GHz (3)	5500	100
	Wi-Fi 5 GHz (4)	5600	100
	Wi-Fi 5 GHz (5)	5700	100
	Wi-Fi 5 GHz (6)	5800	100
IoT	ISM	921.5	35

Bands highlighted in **bold red** were included in the analyses; all other bands were programmed on the ExpoM-RF4 for measurement but were not used in the results presented in this paper.
*Bands programmed in the second ExpoM-RF4 device used for measurements in Australia.
DAB = Digital Audio Broadcasting; UHF DVB-T = Ultra High Frequency Digital Video Broadcasting - Terrestrial; DECT = Digital Enhanced Cordless Telecommunications; DL = Downlink; FM = Frequency Modulation; FDD = Frequency Division Duplexing; ISM = Industrial, Scientific and Medical band; TDD = Time Division Duplex; SDL = Supplementary Downlink; UL = Uplink; VHF – Very High Frequency; WiFi = Wireless Fidelity

Table S11. Smartphone models and network providers used in each country.

Country	Provider	Samsung S22+	Samsung A22	Redmi Note 11S	Oppo A77
France	SFR	✓	✗	✓	✓
	Free Mobile	✓	✗	✓	✓
	Bouygues Telecom	✓	✗	✓	✓
	Orange	✓	✗	✓	✓
Netherlands	KPN	✗	✓	✓	✓
	Odido	✗	✓	✓	✓
	Vodafone	✗	✓	✓	✓
Switzerland	Swisscom	✓	✓	✓	✓
	Sunrise	✗	✓	✓	✓
	Salt	✗	✓	✓	✓
Spain	Movistar	✓	✓	✗	✗
	MasMov	✗	✓	✗	✗
	Vodafone	✗	✓	✗	✗
	Orange	✗	✓	✗	✗
Greece	Cosmote	✓	✓	✗	✗
	Nova	✗	✓	✗	✗
	Vodafone	✗	✓	✗	✗
Germany	Vodafone	✓	✗	✗	✗
Australia	Telstra	✓	✗	✗	✗
	Optus	✓	✗	✗	✗
	Vodafone	✓	✗	✗	✗

Table S12. Activities performed with the phone during measurements in each country.

Country	Non-use	Native voice call	WhatsApp voice call	WhatsApp video call	File upload
France	✗	✓	✓	✓	✓
Netherlands	✗	✓	✓	✓	✓
Switzerland	✗	✓	✓	✓	✓
Spain	✓	✓	✓	✗	✓
Greece	✓	✓	✓	✗	✓
Germany	✓	✓	✗	✗	✓
Australia	✓	✓	✗	✗	✓

Table S13. ExpoM-RF4 band pairs considered for crosstalk correction (see Section 2.4.1).*

	**Band pair**	**Min. – Max. (MHz) Band 1**	**Min. – Max (MHz) Band 2**
Europe	Broadcasting (3) – 700 UL	622.0 – 697.0	700.5 – 735.5
	700 UL – 700 SDL	700.5 – 735.5	730.5 – 765.5
	700 SDL – 700 DL	730.5 – 765.5	753.0 – 788.0
	188 DL – DECT	1805.0 – 1880.0	1880.0 – 1915.0
	DECT – 2100 UL	1880.0 – 1915.0	1919.5 – 1994.5
	ISM 2.4 GHz – 2600 UL	2388.0 – 2488.0	2497.5 – 2572.5
Australia	UHF DVB-T – 700 UL	588.0 – 688.0	682.5 – 757.5
	700 UL – 700 DL	682.5 – 757.5	749.5 – 824.5
	700 DL – 800 UL	749.5 – 824.5	817.5 – 852.5
	800 UL – 800 DL	817.5 – 852.5	855.0 – 890.0
	800 DL – 900 UL	855.0 – 890.0	885.0 – 920.0
	900 UL – ISM	885.0 – 920.0	904.0 – 939.0
	ISM – 900 DL	904.0 – 939.0	932.5 – 967.5
	1800 DL – DECT	1805.0 – 1880.0	1880.0 – 1915.0
	DECT – 2100 UL	1880.0 – 1915.0	1912.5 – 1987.5
	2300 TDD – ISM 2.4 GHz	2300.0 – 2400.0	2400.0 – 2500.0

*Crosstalk correction following the methodology described by Eeftens et al. (2018)

UHF DVB-T = Ultra High Frequency Digital Video Broadcasting - Terrestrial; DECT = Digital Enhanced Cordless Telecommunications; DL = Downlink; ISM = Industrial, Scientific and Medical band; TDD = Time Division Duplex; SDL = Supplementary Downlink; UL = Uplink.

Table S14. Observations for NR serving-cell intervals reported by the ETAIN 5G Scientist app (raw data recorded every second) by activity, serving frequency band, and country.

Activity	NR serving frequency band*	France	Netherlands	Switzerland	Spain	Greece	Germany**	Australia
File upload	FDD bands	0	2236	27646	350	1293	0	2750
	3.5GHz TDD bands	**865**	0	**4114**	**3902**	**3250**	0	114
WhatsApp video call	FDD bands	0	2189	27366	-	-	-	-
	3.5GHz TDD bands	**939**	0	**4340**	-	-	-	-
WhatsApp voice call	FDD bands	0	0	16175	367	1036	-	-
	3.5GHz TDD bands	0	0	0	**3289**	**3015**	-	-
Native voice call	FDD bands	0	0	13674	0	0	0	**40**
	3.5GHz TDD bands	0	0	0	0	0	0	**5**
Non-use	FDD bands	-	-	-	117	820	0	2430
	3.5GHz TDD bands	-	-	-	1815	2814	0	495

*3.5GHz TDD bands included: Band 77 - TDD 3300-4200 MHz, Band 78 - TDD 3300-4200 MHz, and Band 48 - TDD 3550 - 3700 MHz. Other FDD bands included: Band 1 - DL 2110–2170 MHz, Band 3 - DL 1805–1880 MHz, Band 8 - DL 925–960 MHz, Band 28 - DL 758–803 MHz, and Band 14 - DL 758-768 MHz.

** Note that all observations for Germany were registered using LTE technology.

Observations highlighted in bold were not included in the analyses.

A dash (–) indicates that measurements were not conducted under the given scenario.

FDD = Frequency Division Duplexing; TDD = Time Division Duplexing.

Table S15. Locations excluded from map conversion function analyses.

Country	City/Town	Location	Location ID	Provider	Reason for excluding
Germany	Freiburg im Breisgau	Münsterplatz A	DE-DE011	Vodafone	Measurement protocol included only one national provider
	Freiburg im Breisgau	Münsterplatz B	DE-DE012		
	Freiburg im Breisgau	Münsterplatz C	DE-DE013		
	Freiburg im Breisgau	Vauban A	DE-DE021		
	Freiburg im Breisgau	Vauban B	DE-DE022		
	Freiburg im Breisgau	Vauban C	DE-DE023		
	Freiburg im Breisgau	Industriegebiet Nord A	DE-DE031		
	Freiburg im Breisgau	Industriegebiet Nord B	DE-DE032		
	Freiburg im Breisgau	Industriegebiet Nord C	DE-DE033		
	Freiburg im Breisgau	Hauptbahnhof A	DE-DE041		
	Freiburg im Breisgau	Hauptbahnhof B	DE-DE042		
	Freiburg im Breisgau	Hauptbahnhof C	DE-DE043		
	Freiburg im Breisgau	Seepark A	DE-DE051		
	Freiburg im Breisgau	Seepark B	DE-DE052		
	Freiburg im Breisgau	Seepark C	DE-DE053		
Australia	Sydney	Centennial park A	AUS-SD051	Vodafone	Technical recording failure
	Kyneton	High street A	AUS-KY011	Optus	Technical recording failure
	Kyneton	High street B	AUS-KY012	Optus	Technical recording failure
	Kyneton	High street C	AUS-KY013	Optus	Technical recording failure
	Kyneton	Baynton Street	AUS-KY010	Vodafone	Technical recording failure
	Gisborne	New Gisborne A	AUS-GS011	Vodafone	Technical recording failure
	Gisborne	New Gisborne C	AUS-GS013	Vodafone	Technical recording failure

Table S16. Summary of signal strength and environmental exposure for location-level aggregates in Europe and Australia.

	Europe	Australia
Signal strength indicators		
**LTE-RSRP (dBm)**		
Mean (SD)	-82.5 (12.5)	-68.5 (10.1)
Median (Q1, Q3)	-83.0 (-89.5, -72.4)	-67.1 (-75.5, -62.6)
Min. - Max.	-112.3, -58.4	-94.6, -49.0
N	61	48
**LTE-RSSI (dBm)**		
Mean (SD)	-72.2 (11.7)	-
Median (Q1, Q3)	-72.8 (-78.9, -63.6)	-
Min. - Max.	-99.6, -50.7	-
N	61	0
**NR-ssRSRP (dBm)**		
Mean (SD)	-88.2 (14.2)	-
Median (Q1, Q3)	-89.7 (-96.3, -76.5)	-
Min. - Max.	-118.5, -51.6	-
N	48	0
**Combined received power across LTE and NR (dBm)**		
Mean (SD)	-79.8 (11.7)	-
Median (Q1, Q3)	-81.4 (-87.4, -70.6)	-
Min. - Max.	-107.6, -51.6	-
N	48	0
Environmental RF-EMF exposure		
**From LTE (dBμVm^{-1})**		
Mean (SD)	114.9 (10.3)	123.3 (8.3)
Median (Q1, Q3)	104.6 (98.2, 113.5)	119.0 (112.5, 123.0)
Min. - Max.	83.4, 124.9	95.1, 132.7
N	61	48
**From LTE and NR (dBμVm^{-1})**		
Mean (SD)	115.8 (7.6)	-
Median (Q1, Q3)	111.4 (106.6, 115.9)	-
Min. - Max.	90.6, 124.2	-
N	48	0

Values are location-level aggregates used in the map conversion analyses. Signal strength indicators were first aggregated across cells, then averaged per provider and combined across providers within each location, as described in Section 2.4.4. Map conversion analyses were limited to locations where the measurement protocol included all major national network providers. Therefore, sample sizes differ by metric and region, and dashes indicate metrics that were not included in the corresponding map conversion analysis. Combined received power across LTE and NR was calculated as the sum of location-level LTE-RSRP and NR-ssRSRP on the linear scale, back-transformed to dBm. Environmental RF-EMF exposure from LTE networks represents the sum of measured DL and TDD bands below 2.6 GHz. Environmental RF-EMF exposure from LTE and NR networks represents the sum of all measured mobile DL and TDD bands.

dBm = decibel-milliwatts; LTE = Long Term Evolution; NR = New Radio; RSRP = reference signal received power; RSSI = received signal strength indicator; SD = standard deviation; Q1 = first quartile; Q3 = third quartile.

Box S1. Conversion of signal strength or quality indicators to linear scale.

$$P_{mW} = 10^{\frac{P_{dBm}}{10}}$$

$$Q_{lin} = 10^{\frac{Q_{dB}}{10}}$$

where:

- P_{dBm} is the signal strength (e.g. RSRP, RSSI) in dBm and Q_{dB} is the signal quality (e.g. RSRQ) in dB, reported by the ETAIN 5G Scientist app

Box S2. Conversion of electric field strength values to power flux density.

$$S_{mWm^{-2}} = (E_{V\cdot m^{-1}})^2(\frac{10000}{3767})$$

where:

- $E_{Vm^{-1}}$ is the measured electric field strength by the ExpoM-RF4 (in $V\cdot m^{-1}$) and $S_{mWm^{-2}}$ is the power flux density (in $mW\cdot m^{-2}$)

Box S3. Computation of mean signal strength and quality indicators for technical conversion functions.

For each serving-cell interval i, mean signal strength (e.g., RSRP, RSSI) and quality indicators were calculated as:

$$\bar{P}_{lpai} = \frac{1}{n_{lpai}} \sum_{t=1}^{n_{lpai}} P_{lpait}$$

$$\bar{Q}_{lpai} = \frac{1}{n_{lpai}} \sum_{t=1}^{n_{lpai}} Q_{lpait}$$

where:

- $\bar{P}_{lpai}$ is the mean signal strength (expressed in mW) during serving-cell interval i, at location l, for provider p, and activity a
- $\bar{Q}_{lpai}$ is the mean signal quality (expressed in linear scale) during serving-cell interval i, at location l, for provider p, and activity a
- P_{lpait} is the signal strength (expressed in mW) at time point t, during serving-cell interval i, at location l, for provider p, and activity a
- Q_{lpait} is the signal quality (expressed in linear scale) at time point t, during serving-cell interval i, at location l, for provider p, and activity a
- n_{lpai} is the number of time points within serving-cell interval i, at location l, for provider p, and activity a

Box S4. Computation of mean environmental RF-EMF exposure in the serving band for technical conversion functions.

For each serving-cell interval i, mean environmental RF-EMF exposure in the serving band was calculated as:

$$\bar{E}_{lpai} = \frac{1}{n_{lpai}} \sum_{t=1}^{n_{lpai}} E_{lpait}$$

where:

- $\bar{E}_{lpai}$ is the mean measured serving-band exposure (expressed in $mW \cdot m^{-2}$) during serving-cell interval i, at location l, for provider p, and activity a
- E_{lpait} is the measured exposure (in $mW \cdot m^{-2}$) in the serving frequency band at time point t, where the serving band is defined as the DL or TDD frequency band measured by the ExpoM-RF4 whose predefined frequency range corresponds to that of the serving LTE or NR cell reported by the ETAIN 5G Scientist app.
- n_{lpai} is the number of time points within serving-cell interval i, at location l, for provider p, and activity a

Box S5. Computation of location-level signal indicator aggregates for map conversion functions.

Step 1: Aggregate across cells at each recorded time points

For LTE, signal strength indicators were summed across the serving and detected neighbouring cells at each recorded time point:

$$P_{lpt}^{LTE} = \sum_{c=1}^{C_{lpt}} P_{lptc}^{LTE}$$

where:

- P_{lpt}^{LTE} is the aggregated LTE signal strength (in mW) at location l, for provider p, at recorded time point t
- P_{lptc}^{LTE} is the signal strength indicator of cell c, at location l, for provider p, at recorded time point t
- C_{lpt} is the number of LTE cells detected at location l, for provider p, at recorded time point t

For LTE, signal quality indicators were averaged across the serving and detected neighbouring cells at each recorded time point:

$$Q_{lpt}^{LTE} = \frac{1}{C_{lpt}} \sum_{c=1}^{C_{lpt}} Q_{lptc}^{LTE}$$

where:

- Q_{lpt}^{LTE} is the aggregated LTE signal strength (in linear scale) at location l, for provider p, at recorded time point t
- Q_{lptc}^{LTE} is the signal strength indicator of cell c, at location l, for provider p, at recorded time point t
- C_{lpt} is the number of LTE cells detected at location l, for provider p, at recorded time point t

For NR, only serving-cell recordings were considered:

$$P_{lpt}^{NR} = P_{lptc_s}^{NR}$$

$$Q_{lpt}^{NR} = Q_{lptc_s}^{NR}$$

where:

- $P_{lptc_s}^{NR}$ is the signal strength indicator (in mW) of the serving NR cell c_s at location l, for provider p, at recorded time point t
- $Q_{lptc_s}^{NR}$ is the signal strength indicator (in mW) of the serving NR cell c_s at location l, for provider p, at recorded time point t

Step 2: Average across recorded time points to obtain provider-specific values at each location

Provider-specific signal strength and signal quality indicators at each location were calculated by averaging across all recorded time points:

$$\bar{P}_{lp}^{k} = \frac{1}{T_{lp}} \sum_{t=1}^{T_{lp}} P_{lpt}^{k}$$

$$\bar{Q}_{lp}^{k} = \frac{1}{T_{lp}} \sum_{t=1}^{T_{lp}} Q_{lpt}^{k}$$

where:

- k denotes the mobile technology (LTE or NR)
- $\bar{P}_{lp}^{k}$ and $\bar{Q}_{lp}^{k}$ are the provider-specific mean signal strength (in mW) and signal quality (in linear scale) indicators at location l, for provider p
- $T_{lp}$is the total number of recorded time points for provider p at location l

Step 3: Combine indicators across providers to obtain location-level aggregates

Signal strength indicators were combined across providers by summation:

$$P_{l}^{k} = \sum_{p=1}^{N_l} \bar{P}_{lp}^{k}$$

where:

- $P_{l}^{k}\ is$ the location-level signal strength indicator (in mW) for technology k
- N_l is the number of network providers included at location l

Signal quality indicators were combined across providers by averaging:

$$Q_{l}^{k} = \frac{1}{N_l} \sum_{p=1}^{N_l} \bar{Q}_{lp}^{k}$$

where:

- $Q_{l}^{k}\ is$ the location-level signal quality indicator (in linear scale) for technology k
- N_l is the number of network providers included at location l

Composite location-level signal metrics were calculated as:

$$P_{l}^{combined} = P_{l}^{LTE} + P_{l}^{NR}$$

$$Q_{l}^{combined} = \frac{Q_{l}^{LTE} + Q_{l}^{NR}}{2}$$

where:

- $P_{l}^{\text{combined}}$is the combined received power indicator at location l (expressed in mW)
- $Q_{l}^{\text{combined}}$is the combined signal quality indicator at location l (expressed in linear scale)

Box S6.Computation of mean environmental RF-EMF exposure from mobile networks at each location for map conversion functions.

Step 1: Time-averaged exposure per frequency band at each location

For each frequency band b, the mean environmental RF-EMF exposure at location l was calculated as:

$$\bar{E}_{lb} = \frac{1}{T_l} \sum_{t=1}^{n_l} E_{lbt}$$

where:

- $\bar{E}_{lb}$ is the mean exposure (expressed in mW·m^{-2}) in frequency band b at location l
- E_{lbt} is the ExpoM-RF4 measurement (expressed in mW·m^{-2}) in band b at location l, at recorded time point t
- T_l is the total number of recorded time points at location l

Step 2: Environmental RF-EMF exposure from LTE networks

Environmental RF-EMF exposure from LTE networks at each location was defined as the sum of all DL and TDD bands below 2.6 GHz:

$$E_l^{LTE} = \sum_{b \in B_{LTE}} \bar{E}_{lb}$$

where:

- E_l^{LTE} is the total environmental RF-EMF exposure in 4G-LTE bands (expressed in mW·m^{-2}) at location l
- B_{LTE} is the set of DL and TDD frequency bands below 2.6 GHz by the ExpoM-RF4

Step 3: Environmental RF-EMF exposure from LTE and NR networks

Environmental RF-EMF exposure from LTE and NR networks at each location was defined as the sum of all measured DL and TDD bands:

$$E_l^{total} = \sum_{b \in B_{LTE,NR}} \bar{E}_{lb}$$

where:

- E_l^{total} is the environmental RF-EMF exposure from LTE and NR networks (expressed in mW·m^{-2}) at location l
- $B_{LTE,NR}$ includes all measured mobile DL and TDD frequency bands by the ExpoM-RF4

Box S7. Conversion of electric field strength to dBμV·m⁻¹

$$E_{dB\mu V \cdot m^{-1}} = 20log_{10}(E_{V \cdot m^{-1}} \cdot 1e^{6})$$

where:

- $E_{dB\mu V \cdot m^{-1}}$ is the electric field strength in $dB\mu V \cdot m^{-1}$
- $E_{V \cdot m^{-1}}$ is the electric field strength in $V \cdot m^{-1}$

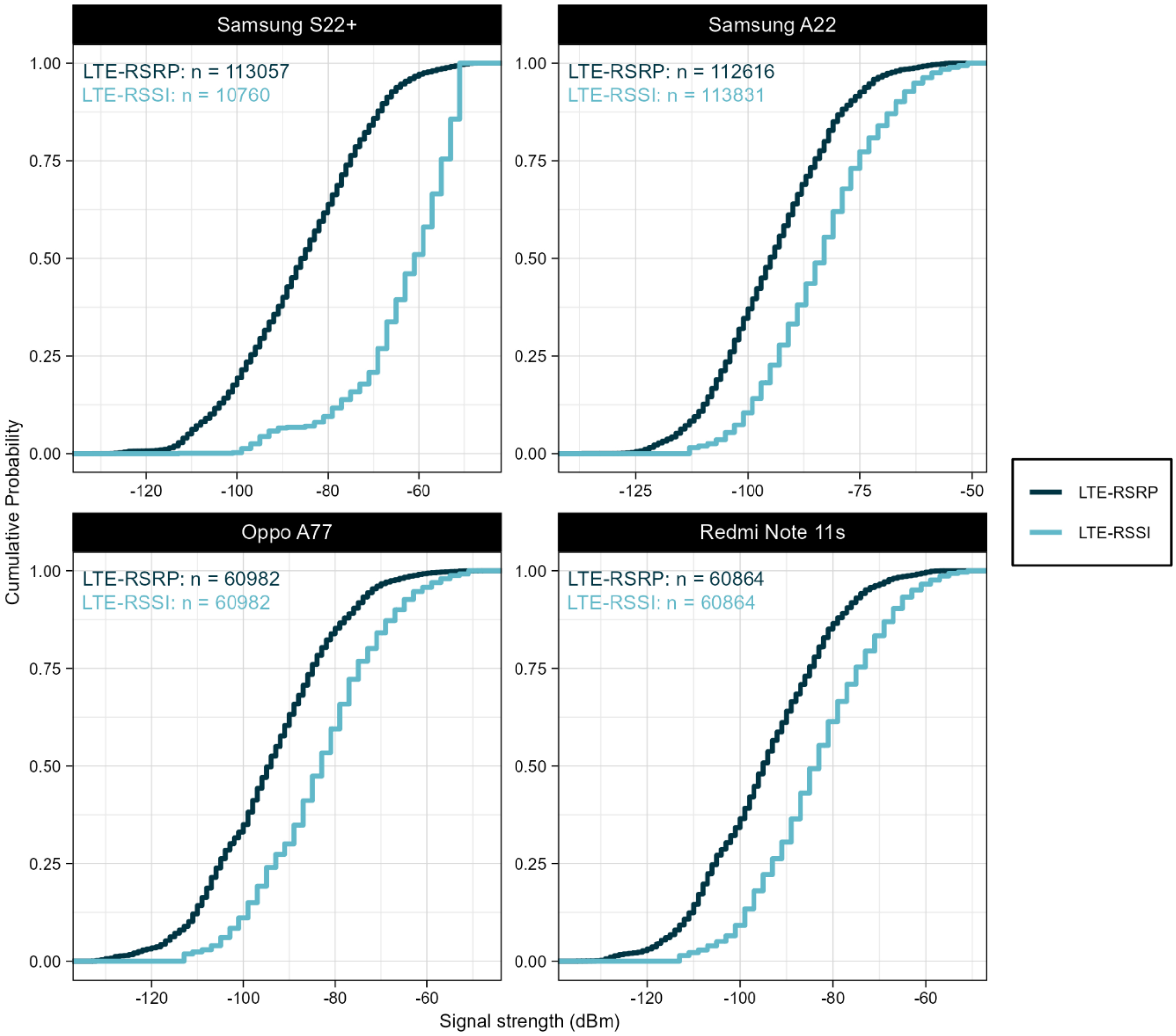

Figure S1. Cumulative distribution functions of LTE-RSRP and LTE-RSSI recorded with the ETAIN 5G Scientist app by smartphone model (raw data recorded every second) for Europe and Australia (data across all countries).

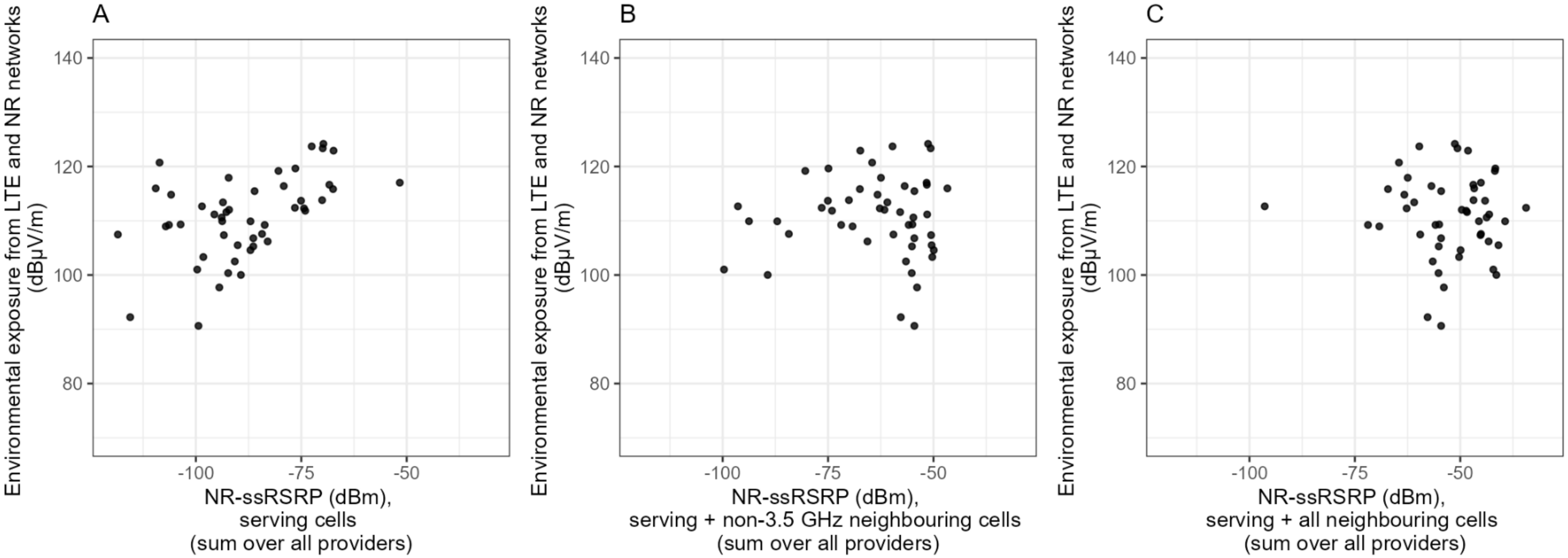

Figure S2. Scatterplots between location-level NR-ssRSRP indicators and measured environmental RF-EMF exposure from LTE and NR networks. Each point represents one spot measurement location. NR-ssRSRP values were first summed across the relevant NR cells on the linear power scale, then averaged across recorded time points within each network provider and location, and finally summed across providers at the location level before conversion back to dBm. Panel A shows NR-ssRSRP based on serving cells only. Panel B shows NR-ssRSRP based on serving cells plus detected neighbouring NR cells operating in bands other than 3.5 GHz. Panel C shows NR-ssRSRP based on serving cells plus all detected neighbouring NR cells.

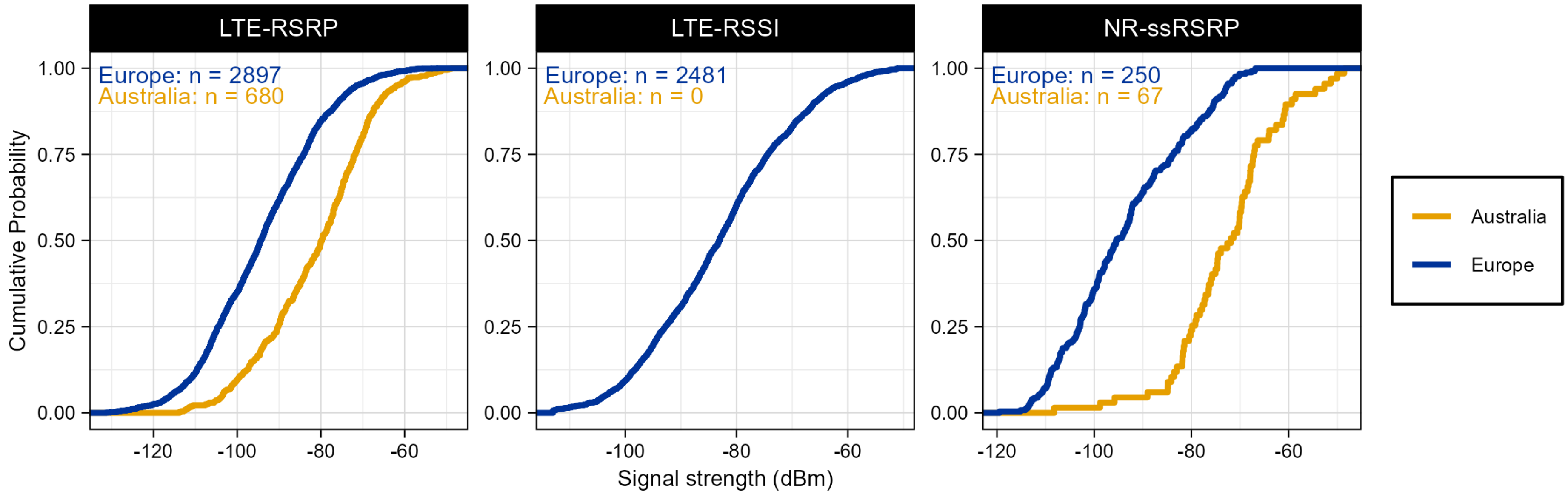

Figure S3. Cumulative distribution functions of serving-cell LTE and NR signal strength indicators in Europe and Australia (serving-cell interval aggregates).

No LTE-RSSI curve is shown for Australia because LTE-RSSI recordings from the Samsung S22+ were excluded due to a device-specific artefact (see Section 2.4.2).

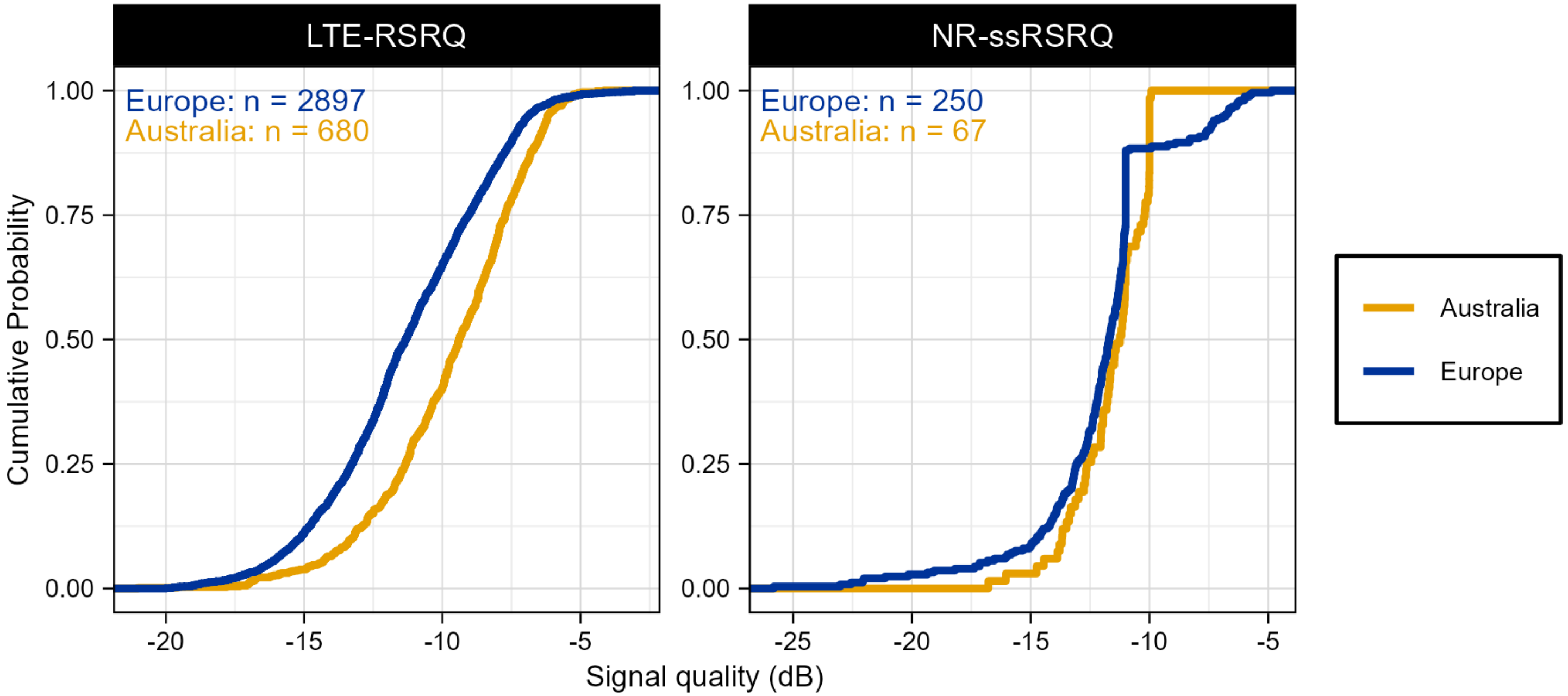

Figure S4. Cumulative distribution functions of serving-cell LTE and NR signal quality indicators in Europe and Australia (serving-cell interval aggregates)

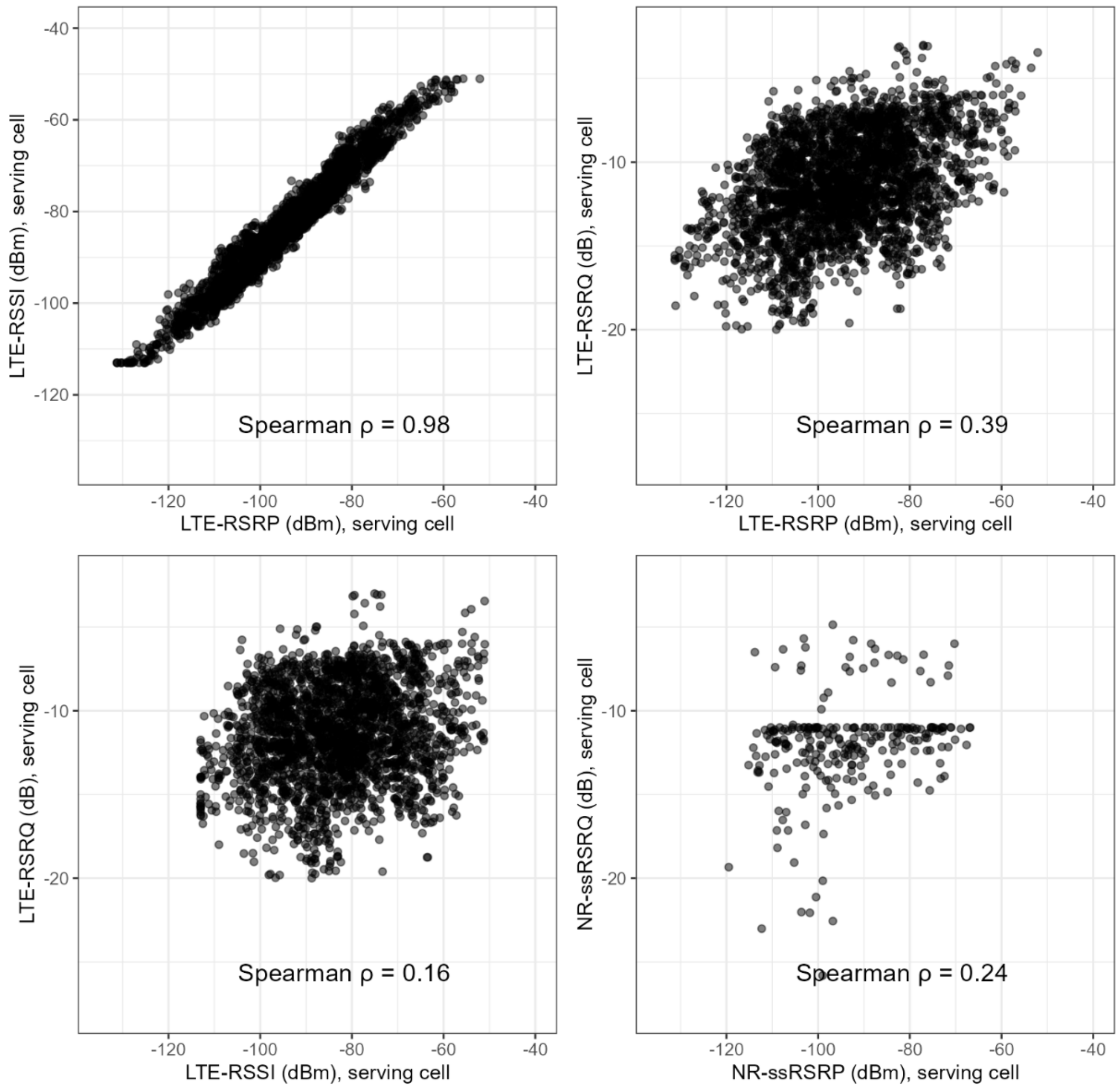

Figure S5. Correlation between signal strength indicators and signal quality indicators collected with the ETAIN 5G Scientist app (data from Europe). Points are plotted with transparency; darker regions indicate areas of higher point density due to overlapping observations.

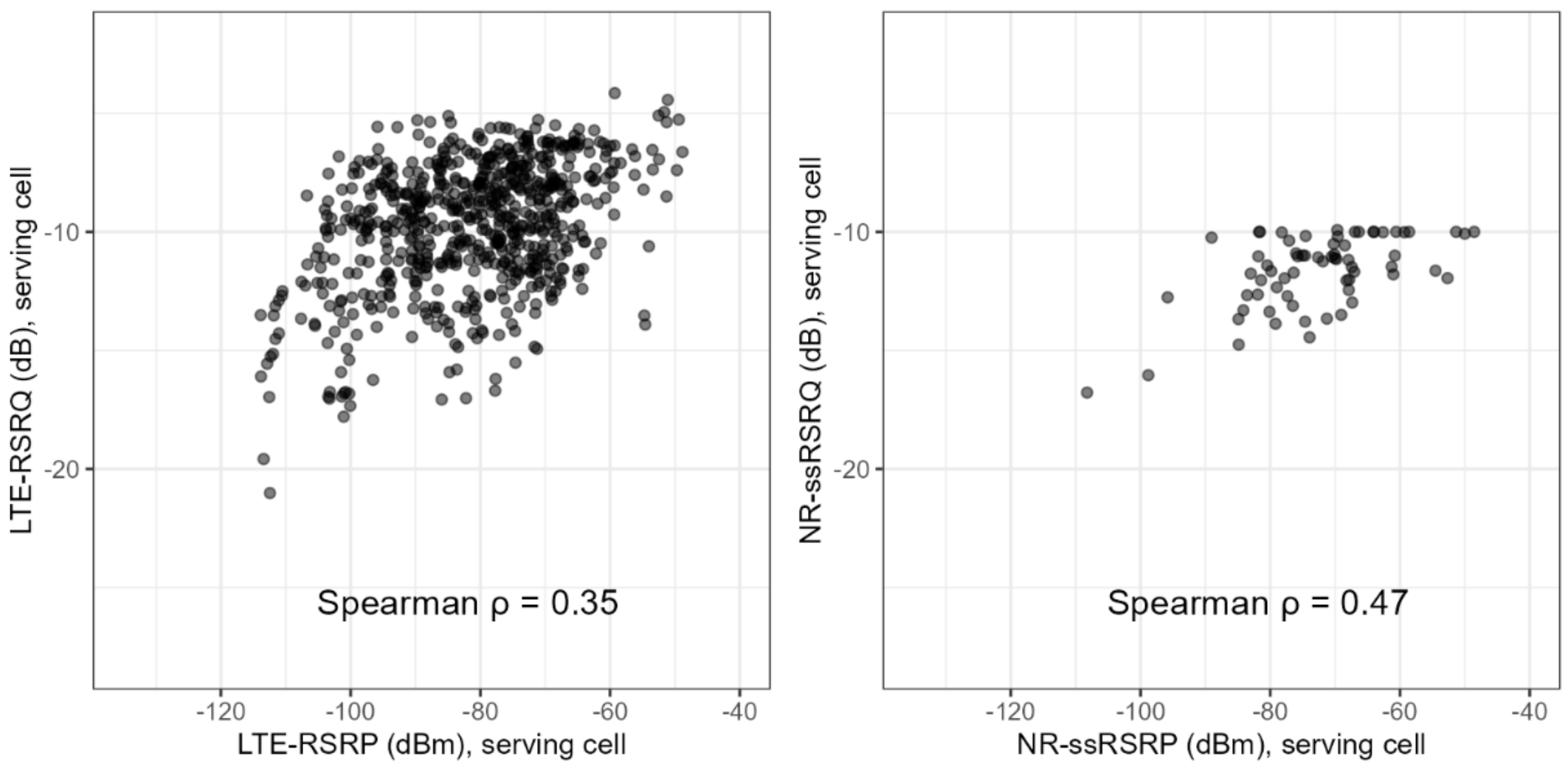

Figure S6. Correlation between signal strength indicators and signal quality indicators collected with the ETAIN 5G Scientist app (data from Australia).
